# Utilizing Collocated Crop Growth Model Simulations to Train Agronomic Satellite Retrieval Algorithms

**DOI:** 10.3390/rs10121968

**Published:** 2018-12-06

**Authors:** Nathaniel Levitan, Barry Gross

**Affiliations:** Department of Electrical Engineering, City College of New York, 160 Convent Ave., New York, NY 10031, USA; gross@ccny.cuny.edu

**Keywords:** crop growth models, MODIS, BLSTMs

## Abstract

Due to its worldwide coverage and high revisit time, satellite-based remote sensing provides the ability to monitor in-season crop state variables and yields globally. In this study, we presented a novel approach to training agronomic satellite retrieval algorithms by utilizing collocated crop growth model simulations and solar-reflective satellite measurements. Specifically, we showed that bidirectional long short-term memory networks (BLSTMs) can be trained to predict the in-season state variables and yields of Agricultural Production Systems sIMulator (APSIM) maize crop growth model simulations from collocated Moderate Resolution Imaging Spectroradiometer (MODIS) 500-m satellite measurements over the United States Corn Belt at a regional scale. We evaluated the performance of the BLSTMs through both k-fold cross validation and comparison to regional scale ground-truth yields and phenology. Using k-fold cross validation, we showed that three distinct in-season maize state variables (leaf area index, aboveground biomass, and specific leaf area) can be retrieved with cross-validated R^2^ values ranging from 0.4 to 0.8 for significant portions of the season. Several other plant, soil, and phenological in-season state variables were also evaluated in the study for their retrievability via k-fold cross validation. In addition, by comparing to survey-based United State Department of Agriculture (USDA) ground truth data, we showed that the BLSTMs are able to predict actual county-level yields with R^2^ values between 0.45 and 0.6 and actual state-level phenological dates (emergence, silking, and maturity) with R^2^ values between 0.75 and 0.85. We believe that a potential application of this methodology is to develop satellite products to monitor in-season field-scale crop growth on a global scale by reproducing the methodology with field-scale crop growth model simulations (utilizing farmer-recorded field-scale agromanagement data) and collocated high-resolution satellite data (fused with moderate-resolution satellite data).

## 1. Introduction

### 1.1. Background

Understanding the effect that environmental and agromanagement factors—such as weather, soil, and fertilization—have on crop growth is a critical question in agronomy-related fields [1]. Several applications—such as adaptation to climate change [2], optimizing agricultural policies [3,4], supporting precision agriculture [5], and reducing yield gaps [6] —require isolating the effect of a particular variable from the other factors affecting crop growth. In order to isolate one of these factors, it is necessary to have good estimates of the other factors in the agricultural system being studied.

Mechanistic crop growth models are well-suited for the task of analyzing the effect that changing a particular factor will have while keeping the other factors constant. This is because they seek to physically model the major genotype, environment, and management (G × E × M) interactions that affect the individual components of the crop-soil system and, ultimately, the yield. Unfortunately, the detailed agromanagement information to run field-scale crop models is, in general, unavailable [3] at a national or global scale, introducing significant uncertainty into the model predicted effect of soil variability, weather variability, irrigation changes, or fertilization changes on the attainable crop yield [6]. Upscaling strategies [7,8] and gridded modeling strategies [2,9,10] have been developed to address the limitation on the availability of data; however, significant uncertainties remain, especially due to limited agromanagement information [11–14]. Gridded modeling strategies can potentially reduce the effects of the limited data availability by calibrating to identify locally optimal crop growth model parameters on a regional scale [15,16]. Unfortunately, these studies have been limited by generally only using regional yields for calibration. For example, in [16], two unknown G × E × M factors (the planting date and planting density) and three crop growth model coefficients (the biomass to energy ratio, the harvest index, and the potential heat units) are calibrated based only on goodness-of-fit criteria with United State Department of Agriculture National Agricultural Statistics Service (USDA-NASS) county-level maize yields. Further, even this calibration with regional crop yields is not always performed [10], likely because the stresses imposed on crop growth, especially in developing regions, are highly variable and dependent on unknown field-scale management decisions.Gridded crop models perform significantly worse in developing regions [10].

**Figure 1.**
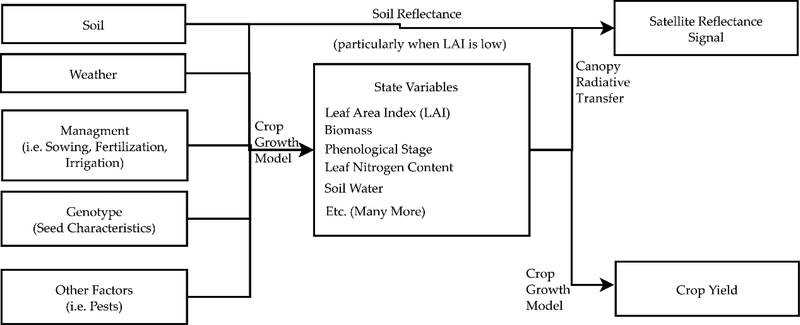
Interrelationships among yield, satellite measurements, crop state variables and G × E × M factors.

Satellite remote sensing provides an alternative to these two approaches to mitigate the effects of limited data availability because it makes field scale measurements with global coverage. As the atmospherically-corrected satellite reflectance signal is strongly affected by the in-season crop state [17], it provides the potential to efficiently collect a large dataset on crop-soil system state variables. The state variables describe the dynamic evolution of the plant structure and soil state in time and encompass variables such as the leaf area index (LAI), aboveground biomass, phenological stage, and soil moisture. Canopy radiative transfer (RT) models [18] provide the theoretical basis that links the state variables and soil reflectance (which is very influential when LAI is low [19,20]) with the satellite reflectance signal. Field measurements of these in-season state variables are greatly beneficial in the calibration of field-level crop growth models [21–23] and it can thus be supposed that a high resolution global dataset of in-season state variables can also improve the calibration of regional crop growth models. As illustrated in Figure 1, the in-season state variables of a crop are a function of the G × E × M factors (both physically and in crop growth models) and, as a result, similar to the efforts in [15,16], a calibration of a regional crop growth model with these in-season state variable measurements would represent a reduction in the uncertainty of some of the unknown G × E × M factors and the crop growth model coefficients that describe their effect on crop growth. As seen in Figure 1, a mechanistic crop growth model calculates the yield from the in-season state variables, so the connection between the G × E × M factors and the state variables is more direct than that of the yield. This explains why performing multi-objective calibration against both the state variables and yields can result in models that outperform those calibrated against yields alone.

The difficulty in directly using the satellite reflectance signal to retrieve the state variables is that the connection between the state variables and the satellite reflectance signal is very complex. The large number of inputs to canopy RT models [18] makes their inversion highly ill-posed, especially as one attempts to retrieve more than one variable [24,25]. Because of the limited availability of data about the distribution of canopy vegetation characteristics, studies must assume wide ranges of the unknown canopy RT model inputs when performing the inversions [26], limiting the quality of the results. Complex algorithms have been developed to invert the canopy RT models to retrieve the LAI and leaf chlorophyll content in maize [19]; however, significant uncertainties remain. Furthermore, when coupling canopy RT and crop growth models, significant further uncertainty is introduced because the variables that are inputs to canopy RT models are not necessarily the same as those outputted by crop growth models [17,27–29]. Some of these variables can be coupled with empirical relationships. For example, crop growth models generally output the leaf nitrogen content, which can be converted to the leaf chlorophyll content inputted to canopy RT models by an empirical relationship, such as that in [30]. However, the leaf nitrogen-chlorophyll relationship in [30] only showed an R^2^ value of 0.73 and was validated using only data from an N-rate trial at a single location in Shelton, Nebraska in 2006. Other inputs, such as the average leaf angle and the equivalent water thickness, must simply be assumed to be constants. Empirical approaches to retrieve the canopy vegetation characteristics and crop growth state variables, trained with both proximal and collocated remote sensing, are also fraught with large uncertainties due to limited data availability that causes the empirical models to generalize poorly to new environments [31], even after nearly 30 years of data collection and research. Reducing the uncertainties in retrieving canopy vegetation characteristics and in the coupling of canopy RT models and crop growth models would thus require extensive field campaigns in a wide range of G × E × M environments using traditional methods; the expense of carrying out such extensive field campaigns calls for new approaches.

A promising line of research in agronomic satellite retrieval algorithms, which has recently seen new interest [32–34], has been crop-model-based regression (CM-Reg), which was first introduced by [35]. CM-Reg generates a large, synthetic ensemble of crop model simulations and corresponding vegetation indexes, either simulated by the crop model itself [35] or estimated via empirical relationships from its outputs [32–34]. CM-Reg then uses this synthetic ensemble to estimate an empirical relationship between the crop model-simulated yield and simulated vegetation indexes. This empirical relationship, determined solely from synthetic crop model simulations, is then used to predict actual yields from actual satellite measurements. While CM-Reg does not [34] necessarily produce significantly better results than simpler empirical approaches [36,37] that regress satellite measurements against regional surveyed yields, it has a significant advantage in that it is more generalizable to new environments. This is because its yield prediction is based on mechanistic crop model simulations, rather than being purely empirical [34]. However, as the ensemble used by CM-Reg to determine a relationship between the crop yields and vegetation indices is synthetic, CM-Reg’s power will always be limited by the need to predict vegetation index time series for the synthetic simulations. Our work takes inspiration from CM-Reg to address the concern of limited data availability to train agronomic satellite retrieval algorithms and provides a framework to increase the number of variables retrieved.

### 1.2. Overview

As stated in Section 1.1, the need for extensive field measurements to calibrate canopy RT models and their coupling with crop growth models serves as a major roadblock in fully utilizing satellite measurements to calibrate regional crop growth models. In this study, we explore whether utilizing collocated crop growth model simulations and satellite measurements can serve as an alternative to utilizing ground measurements in training agronomic satellite retrieval algorithms. This approach is similar to CM-Reg in that it seeks to learn an empirical relationship between crop growth simulation output variables and satellite measurements from a database of these quantities. However, by utilizing crop growth model simulations that perform accurate predictions at fixed locations and are not synthetic, our method is able to use the actual satellite measurements to remove the major limitation of CM-Reg that one must have a method to calculate synthetic satellite measurements from the crop model simulations. Obtaining synthetic satellite measurements for synthetic crop model simulations is fraught with difficulties, as seen by the challenges experienced [17,27–30] in coupling crop growth models to canopy RT models. This indicates that replacing the synthetic satellite measurements with actual measurements would provide a very interesting enhancement to CM-Reg.

**Figure 2.**
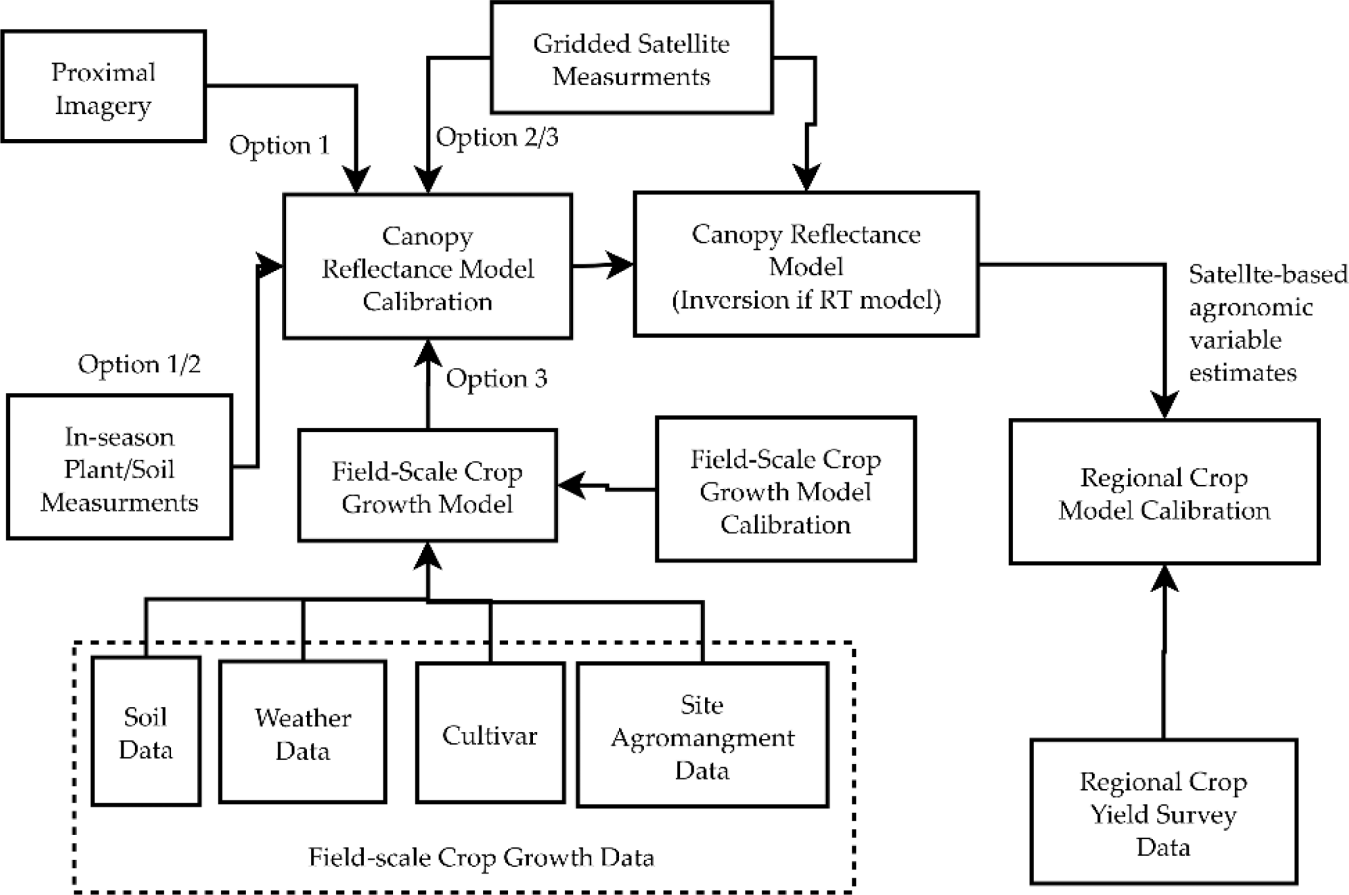
Flowchart of proposed method of calibrating canopy reflectance models as compared to traditional options. The traditional options (1 and 2) use collocated in-season plant/soil measurements along with proximal or satellite imagery, while the proposed Option 3 uses field scale crop growth simulations in place of the in-season measurements. The calibrated canopy reflectance models can be used in the future for regional crop model calibration.

Our method to utilize collocated crop growth model simulations and satellite measurements is outlined in Figure 2 as Option 3, along with more traditional approaches to crop remote sensing, which we label as Options 1 and 2. The traditional options (Options 1 and 2) use collocated imagery and physical measurements of the in-season state variables and yields to calibrate canopy reflectance models; Option 1 uses proximal imagery, while Option 2 uses collocated satellite imagery. In contrast, Option 3 uses collocated crop growth model simulations to replace the physical measurements, allowing for the development of algorithms to retrieve the variables that do not have field measurements.

All three options in Figure 2 lead to the calibration of a canopy reflectance model, which then can be used operationally in the future to obtain estimates of agronomic variables (the yields and in-season state variables) from satellite measurements. Once operational, these estimated in-season state variables and yields can be used to calibrate regional crop models in addition to using the surveyed regional crop yields, following a method similar to that used for only the surveyed yields by [15,16].

It is important to note that there are a variety of methods (and combinations of these methods) that can represent calibration of the canopy reflectance model in Figure 2, such as:

- Optimization of unknown canopy RT model inputs (such as the average leaf angle)
- Optimization of the empirical relationships between crop growth model outputs and canopy RT model inputs
- Optimization of empirical canopy reflectance models that bypass the canopy RT models

In this study, we chose to optimize an empirical model that bypasses the canopy RT models, which allows the satellite measurements to be directly used to obtain estimates of the agronomic variables after optimization. In contrast, if a canopy RT model had been calibrated instead, an inversion method would have had to be applied to estimate the agronomic variables from the satellite measurements and calibrated canopy RT model.

We seek to demonstrate the feasibility of Option 3 as an alternative to Options 1 and 2 because of the difficulties [31] in collecting sufficient in-season plant/soil measurements collocated with imagery to reach an acceptable level of uncertainty with these two traditional options.

The feasibility of Option 3 ultimately rests on the hypothesis that:

1. Accurate, geolocated agromanagement data collected by farmers, supplemented by publicly available high-resolution weather and soil datasets, can be used to provide decent estimates of the water and nitrogen-limited attainable state variables at a set of training sites.
2. In highly developed cropping systems, such as those in the US Corn Belt, the gap between the attainable yields and the actual yields, which have been further reduced by weeds, pests, and other factors, is sufficiently small that significant information about the attainable state variables is contained in the actual state variables.
3. Crop model-predicted state variables at a set of training sites with accurate, geolocated agromanagement data can be used to teach a bidirectional long short-term memory network (BLSTM) to retrieve the attainable state variables solely from the satellite measurements.

Numerous studies [21,38–41] have been devoted to testing the first portion of the hypothesis; particularly notable out of these are the more recent studies [21,38] with the Agricultural Production Systems sIMulator (APSIM) maize model used in this study, which show very strong results in the prediction of the LAI, biomass, leaf nitrogen, soil nitrogen, and soil water time series. Based on these studies, we believe that it is reasonable to assume that the first portion of the hypothesis is true for the purposes of this study. Therefore, in this study, we seek to show that it is reasonable to believe that the second and third portions of the hypothesis are also true. We do so by performing a regional calibration of the APSIM crop growth model across the entire United States Corn Belt at the county-level with USDA-NASS survey data and high-resolution soil and weather data sources. A verification of the ability of APSIM simulations of attainable yields to predict actual surveyed yields serves as a test on whether the magnitude and variability of the yield gap is sufficiently small in the United States Corn Belt that the random variability caused by weeds and pests does not prevent crop growth models from accurately simulating the in-season growth processes on commercial farms that determine the yield. Previous studies, such as that by [11], indicating the capability of crop growth model-simulated attainable yields to predict actual yields over the US Corn Belt, provide support for this ability. We then examine whether county-averaged 500-m Moderate Resolution Imaging Spectroradiometer (MODIS) satellite solar reflectance measurements can predict the calibrated APSIM-predicted attainable in-season state variables with a BLSTM.

## 2. Materials and Methods

### 2.1. APSIM-Maize

As mentioned in Section 1.2, we chose to use the APSIM-maize [42,43] crop growth model to perform the regional-scale simulations for this study based on strong recent validations of its ability to simulate in-season growth processes in the midwestern United States [21] and Queensland, Australia [38]. The APSIM-maize crop growth model was designed as a mechanistic, field-scale crop model that is able to simulate water and nitrogen-limited growth with detailed modeling of soil processes. The soil processes are compartmentalized into a separate APSIM module that is used for several different crops. The soil module is based on a heritage [42] from the Crop Environment Resource Synthesis (CERES) and Productivity, Erosion, and Runoff Functions to Evaluate Conservation Techniques (PERFECT) models and has a cascading bucket style water-balance component, along with modules describing the movement of nitrogen and other nutrients through the soil. Although the modeling of maize growth in APSIM was originally based on the CERES-maize module, APSIM has integrated all crop modeling into a generic crop model template with specific parameters for each crop [44]. The generic crop model template is broken down into seven components (phenology, biomass, canopy, root system, senescence pools, water, and nitrogen) that simulate the growth of the particular crop with crop-specific parameters. Further details on the operation of each crop growth component can be obtained from [44]. Although APSIM-maize is a field-scale crop-growth model, it, as others, has been applied at the regional [45] and global [10] scale.

The sensitivity of APSIM-maize to its model inputs is heavily affected by the environment in which the crop is being grown; a recent study [13] of the sensitivity of APSIM-maize found large variability in the sensitivity index with respect to sowing date and hybrid choice across a range of environments in New Zealand. The results show that different regions expose the crop to different types of environmental stress and the effect caused by changing input parameters depends on the types of stresses present in the environment. However, overall the soil properties, cultivar selection, and management practices are very important to yield; a study [46] with APSIM-maize in Northeast China found that yields can be increased by 9% by improving soil physical properties, by 23% by changing cultivars, and by 34% by improving management practices.

The data input requirements to perform a simulation with APSIM and the sources of the data are listed in Table 1. The data sources are further described in Section 2.2 and the calibration procedure used to determine some of the maize input variables is described in Section 2.3.1. As outlined in Table 1, in order to use county-averaged soil survey data in place of detailed soil sampling, the unavailable APSIM soil inputs were filled utilizing both the procedure in [21] and some APSIM default values. The variable names of the data in the original data sources are listed in parenthesis after in Table 1.

### 2.2. Data

To conduct this study, we obtained collocated soil [47], meteorological [48,49], satellite solar reflectance [50], and USDA NASS survey data for rainfed maize in the United States. Only rainfed maize is considered to avoid the uncertainty introduced by the unknown irrigation amount and application schedule, which can be significant; for example, a study in Northwest China [51] indicated that inequities of water delivery in irrigated areas can cause up to 35% differences in yields. All data was stored in a common MySQL database after the soil and metrological data were reprojected to the resolution and grid of the satellite pixels using gdalwarp [52]. Pixels were selected for this study if they were more than 90% covered by maize for that year, as defined by the appropriate USDA NASS Cropland Data Layer [53], and if they were in counties where less than 10% of the maize is irrigated per the 2012 USDA Farm and Irrigation survey. The gdalwarp averaging function was applied to the USDA NASS Cropland Data Layer to determine if a pixel was more than 90% covered by maize. All qualifying pixels in MODIS tiles h11v04, h11v05, h10v04, and h10v05, which cover the vast majority of maize production in the United States, were included for the years of interest.

As this study was conducted at the county level, the soil, meteorological, and satellite data were averaged within each county over all qualifying pixels covered with maize; only maize pixels were considered to compute the county-level averages. Data from 2008, 2009, 2010, 2011, 2013, 2014, 2015, and 2016 were used to perform the county-level analysis and the spatially averaged time series were stored in MySQL by county-year. A county-year includes all the meteorological, satellite, and USDA NASS survey data necessary for the analysis of a particular county in a particular year. Data from 2012 was not included because of the especially extreme drought, termed a “once-in-a-generation crop calamity” [54], that occurred in the United States Corn Belt during this year which could result in unrepresentative growing conditions unsuitable for this study.

#### 2.2.1. Soil Data

The national Probabilistic Remapping of SSURGO (POLARIS) [47] dataset of gridded soil properties at 30-m resolution, which is based on a state-of-the-art machine learning based interpolation of the USDA Soil Survey Geographic (SSURGO) Database is used to obtain the necessary soil inputs for the APSIM crop growth model, as outlined in Table 1.

#### 2.2.2. Meteorological Data (PRISM and NASA POWER)

The 4-km Parameter-elevation Relationships on Independent Slopes Mode (PRISM) [48] meteorological dataset, based on a state-of-the-art advanced interpolation of weather station data, is used to obtain the daily maximum temperature, daily minimum temperature and daily precipitation. The daily incoming solar radiation, a critical meteorological variable for crop growth models, is not available in the PRISM dataset and is in general difficult to obtain at high spatial resolution due to lack of measurements, although some early stage attempts have been made [55]. As a result, the 1-degree daily incoming solar radiation data from the National Aeronautics and Space Administration Prediction Of Worldwide Energy Resources (NASA POWER) [49] dataset is used and is linked to the county-years in MySQL by nearest-neighbor interpolation. Despite the coarse resolution, the NASA POWER solar radiation dataset has been found to be well-correlated with ground station data and has been assessed as suitable for regional studies [56,57].

#### 2.2.3. Satellite Solar Reflectance Data (MODIS)

The 500 m MODIS MCD43A4 V006 Nadir BRDF-Adjusted Solar Reflectance product [50] is obtained for the pixel-years of interest in seven bands (620–670 nm—red; 841–876 nm—near-infrared; 459–479 nm—blue; 545–565 nm—green; 1230–1250 nm—near-infrared; 1628–1652 nm—short-wavelength infrared, and 2105–2155 nm—short-wavelength infrared) at 8-day intervals, which is appropriate considering the 16-day retrieval period used to generate the product. Satellite data from Julian day 109 to 333 (19 April to 29 November in non-leap years), which corresponds to 29 measurements per county-year, was considered for the satellite retrieval analysis in order to include the entire growing season for all sites (with decent margins for the vast majority of sites). The dates were selected as the earliest and latest eight-day periods from 15 February with maize growth process active among the APSIM simulations. Because raw MODIS measurements are taken with a 1–2 day revisit time for the entire Earth on both the Terra and Aqua platforms and as all measurements in a 16-day period centered on the retrieval date are considered by the MCD43A4 product to perform the BRDF-adjusted surface reflectance retrieval, there are very few gaps in the data caused by clouds and other factors and all 29 surface reflectances were available for >85% of county-years. The small percentage of county-years that were missing retrievals generally did not have more than 1 or 2 retrievals missing out of the 29 and these were filled by linear interpolation in time.

#### 2.2.4. USDA NASS Survey Data

The USDA NASS county-level Survey Crop Yields and state-level Crop Progress Survey data are obtained from 2008–2016 for all counties included in the MySQL database for the analysis. The state-level Crop Progress Survey reports the percentage of fields that have reached a particular phenological stage on a weekly basis. Data for four phenological stages (planting, emergence, silking, and maturity) are used in this study. The data for planting is used an input for the APSIM model, the difference between the maturity and emergence date (i.e., the length of the season) is used to calibrate the APSIM model, and the emergence, silking, and maturity dates are used to validate both the satellite retrieval results and the APSIM simulations.

### 2.3. Methods

#### 2.3.1. APSIM Calibration

In order to obtain representative estimates of county-level variables, it is necessary to calibrate the APSIM-maize module to accurately represent yields and phenological dates across the US Corn Belt. In this calibration procedure, we assume that each region can be represented by a weighted average (i.e., a distribution) of crop model simulations with different agromanagement parameters. Calibration is performed against both the county-level ground-truth USDA NASS crop yields and interpolated state-level growing season lengths, which we define as the number of Julian days between the median emergence and maturity dates from the USDA NASS crop progress report. Two different types of calibration were performed in this study. First, a calibration over the entire US Corn Belt with a constant distribution of agromanagement parameters is performed. Second, a calibration dividing the United States into weather-based clusters and determining a separate distribution of agromanagement parameters for each cluster is performed. The two different calibration approaches are used to robustly explore the remote sensing retrieval approach in light of the tradeoffs inherent in the calibration process. The first approach has a strength in that assuming a constant distribution of agromanagement parameters reduces the likelihood that the model calibration will be overfit because of the significant reduction in the degrees of freedom (i.e., lack of spatial dependence of model parameters). In contrast, the second approach is strong in that it reflects farmer adaptation to the local environment by calibrating different model parameters in different regions, similar to other regional calibration approaches [15,58] in which clusters are determined and agromanagement parameters are assumed to be constant for each cluster. While complex agroecological zones are often used to define the clusters based on the climate, soil, and terrain characteristics [58], in this study we choose to perform a simple k-means clustering on the monthly average daily minimum temperature, daily maximum temperature and precipitation (for all the years of the study period) to define our regions with constant agromanagement parameters. We chose this approach over a more complex clustering that considered both weather and soil properties due to the danger of overfitting the model calibration. By not including soil information in the clustering, the soil component of APSIM is better tested when faced with intracluster soil variability. We also performed calibration on clusters based purely on geographic proximity to further analyze the performance of the calibration.

Calibration was performed on the planting density, seed variety, nitrogen applied, and planting date. Because of the large number of county-years considered (~5000) and the consequent computational cost, only discrete values of these parameters were considered and they are listed in Table 2. The seed variety is broken down into the seed brand and seed relative maturity in Table 2; seeds from different brands with the same relative maturity differ in that they have the same growing degree day values from planting to maturity, but different growing degree day values from planting to flowering. APSIM simulations were run for all combinations of the parameters listed in Table 2 for each county-year with the appropriate soil and weather data.

Because APSIM simulates attainable yields, the APSIM yields were reduced by 15% prior to calibrating them against the NASS actual yields to account for the yield gap caused by pests, weeds, and other factors. Previous studies [60–62] have noted the necessity of making a reduction for these factors when calibrating regional crop models against actual yields. It has been estimated that the gap between attainable yields and actual yields ranges from 20% to 30% over the US Corn Belt [63]; however, a value of 15% was chosen for the adjustment based on [11], which found a 16% average difference between simulated attainable maize yields and actual yields at the national level across the US Corn Belt. As explained by [11], crop models seem to slightly underestimate the attainable yield because they miss some factors that can cause yields to increase; [11] provided an example of water supply from perched water tables as a factor that increases yields that is not captured by the crop model. Another possible factor that can cause underestimation of the attainable yield in this study is the use of only generic cultivars (Table 2) as the discrete options for calibration, which may not be as well adapted to the local environments in the United States as some of the actual cultivars used by farmers. While the 15% value is significantly smaller than the 40% value identified for maize across the US Corn Belt by calibration in [60], we believe that the more recent results in [11] are more physical because they are closer to the observed yield gap [63] and because the model inputs chosen by [11] as more realistic than those chosen by [60] ([11] uses county-specific cultivars, planting dates, and planting densities, while constant values are assumed by [60]). While further research may determine a more accurate value for this adjustment, we believe that the 15% value is reasonable for the current study based on [11] and the 20–30% yield gap observed across the US Corn Belt [63].

To find the best distribution of the agromanagement parameters for each cluster (or over the entire United States for the clusterless calibration), each of the 288 different possible combinations of the parameters in Table 2 were assigned a weight ranging between 0 and 1 by the calibration. It was assumed that the simulations could be used to make predictions of the continuous variables as

(1) $$X_{c}=\sum_{i=1}^{288}w_{i}X_{i},$$

where *X*_*c*_ is the value of the continuous variable predicted by the calibrated model, *w*_*i*_ is the weight for the *i*th combination of agromanagement parameters, and *X*_*i*_ is the value of the corresponding APSIM simulated variable. *X*_*c*_ and *X*_*i*_ can represent yields, phenological dates, and continuously valued in-season state variables (i.e., all those except the discrete phenological stage). The weights were constrained to sum to 1 to ensure that each weight had a physical meaning as the fraction of fields in the cluster that were grown with these agromanagement parameters.

Special treatment is needed for the phenological stage, which is a discrete number. Therefore, we instead use the interpretation of the weights to calculate the fraction of fields in the *m*th phenological stage on day *d* in a county as

(2) $$C_{p}[m,d]=\sum_{i=1}^{288}w_{i}h\left(C_{i}[d],m\right),$$

where *C*_*p*_ is the predicted percentage of fields, *C*_*i*_[*d*] is the phenological stage of the *i*th combination of agromanagement parameters and *h*(*n*, *m*) is defined as

(3) $$h(n,\;m)=\begin{array}{l} 0,n\neq m\\ 1,n=m \end{array}\;,$$

Optimization was performed by minimizing the weights according to

(4) $$\min\left[\frac{{\left(Y_{m,k}-\sum_{i=1}^{288}w_{i}Y_{i,k}\right)}^{2}}{{}^{\sigma }Y^{2}}+\frac{{\left(P_{m,mat-eme,k}-\sum_{i=1}^{288}w_{i}P_{i,mat-eme,k}\right)}^{2}}{{\sigma_{P_{mat-eme}}}^{2}}\right]\;subject\;to\;\begin{array}{c} \sum_{i=1}^{288}w_{i}=1,\;and\\ 0\leq w_{i}\leq 1 \end{array}\;,$$

where *Y*_*m*,*k*_ is the ground-truth USDA NASS crop yield, *P*_*m*,*mat*−*eme*,*k*_ is the ground-truth USDA NASS growing season length (number of Julian days between maturity and emergence dates) interpolated to the county level, *σ*_*Y*_ is the standard deviation of the ground-truth crop yields, *σ*_*P*_*mat*−*eme*__ is the standard deviation of the ground-truth season length, and *Y*_*i*,*k*_ and *P*_*i*,*mat*−*eme*,*k*_ are the APSIM simulated yield and APSIM simulated growing season length for the *i*th combination of agromanagement parameters and *k*th county-year in the cluster. In order to ensure that *P*_*m*,*mat*−*eme*,*k*_ calibrates the simulations to the correct conditions for the county, linear geographic interpolation of all state-level data to the county-level is used in calibration; however, the performance in Sections 3 and 4 is analyzed by averaging to the state level. The standard interior point constrained least square optimization algorithm in MATLAB 2017a (lsqlin) is used to perform the optimization.

As in any calibration procedure, validation is critical to assess model performance and ensure that overfitting has not occurred. Following the procedure in [64], leave-one-out (LOO) cross-validation is used to analyze the performance of the calibration. Specifically, as in [64], the simulation for each county-year is obtained by optimizing the calibration weights *w*_*i*_ with all county-years that are neither of the same year or of the same county as the one being simulated. As a result, the yield predicted by each simulation reflects the skill of the model without any knowledge of the conditions in the current year or current county, providing a strong test on the model’s predictive ability. The LOO coefficient of determination (R^2^) and the root-mean square error (RMSE) are used as the metrics to quantify the model performance. The LOO R^2^ values are calculated at the regional levels, while the LOO RMSE values are calculated at both the regional and county levels. For the spatial analysis, *LOO RMSE*_*County*_ is expressed as the percentage (%) of the overall yield standard deviation (*σ*_*Overall*_) over the entire US Corn Belt, which we term the explained standard deviation (ESTD) and define it as

(5) $$ESTD=100\times \left(1-\frac{LOO\;RMSE_{County}}{\sigma_{Overall}}\right)[\%].$$

The ESTD is reported in place of *LOO RMSE*_*County*_ because it is difficult to interpret RMSE values and compare them to other studies as the magnitude of a cross-validated model’s error depends on the variability (standard deviation) of the actual yields predicted (if the model is calibrated by LOO cross-validation on a dataset where the variability of actual yields is low, the RMSE values will be low even if the model performance is weak). Because the ESTD compares the *LOO RMSE*_*County*_ to the standard deviation of the yield over the entire dataset, it can be used to evaluate the spatial performance of the model as the prediction error in each county is compared to the dataset’s overall yield variability. In contrast, the LOO R^2^ is based on the average error over the regional scale and thus is more difficult to use to evaluate the spatial performance of the model.

Lastly, in order to validate the spatial performance of the model and separate it from its interannual temporal performance, an empirical orthogonal function-based (EOF) model validation analysis is conducted [65,66]; the details of this analysis are included in the Supplementary Materials.

#### 2.3.2. Retrieval of Predicted State Variables from Satellite Measurements

Once the state variables have been predicted by the calibrated APSIM models with Equations (1) and (2), we train BLSTMs to predict the state variables from the county-averaged MODIS measurements. A long short-term memory network (LSTM) is a form of a recurrent neural network that takes a multivariate time series as an input and predicts another multivariate time series as an output; LSTMs have found wide applications due to their strong ability to perform supervised learning in the time domain [67]. The variant of LSTMs that we are using in this study, BLSTMs [68], have the advantage of being able to make predictions with information from both the future and the past due to their bidirectional nature.

**Figure 3.**
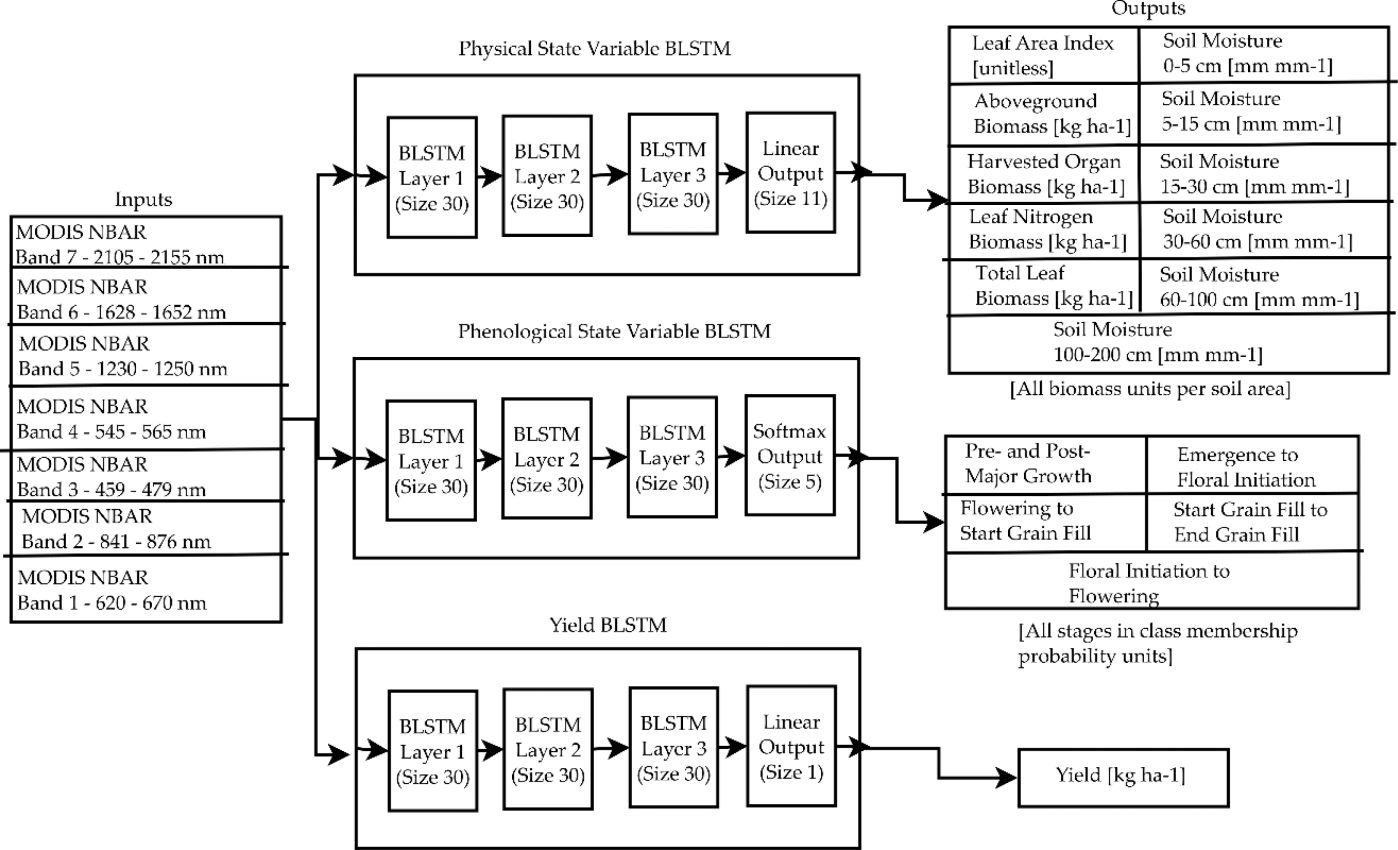
Architecture of BLSTMs used in study. Three separate BLSTMs are used to predict the physical state variables, phenological state variables, and yield from the satellite measurements. The layers of the BLSTMs, according to the definitions used in CURRENNT, are shown for each BLSTM.

A diagram of the BLSTMs used in this study are shown in Figure 3. All three BLSTMs are common in that they all have three BLSTM layers of 30 units each; this deep structure aids the BLSTM in capturing the different time scales of the various processes present in crop growth [69]. The spectral surface reflectances are directly inputted to all three BLSTMs without converting to any vegetation indexes to allow the BLSTM to itself determine the best transformations of the data necessary to perform the retrievals. The BLSTMs are trained using the Munich Open-Source CUDA RecurREnt Neural Network Toolkit (CURRENNT) [68] after the data is extracted from the MySQL database. The layers of the networks and their interconnections, which are inputted to the CURRENT toolkit in a JSON file, are illustrated in Figure 3. All trainable layers have bias values of 1.

Different BLSTMs are used for the different types of variables for the following reasons:

- The physical state variable-predicting BLSTM uses a standard linear output layer and sum of square errors cost function. Each of the physical state variables is normalized to zero mean and unit variance using the training data to ensure that units do not cause the network to favor training one of the state variables over another.
- The yield-predicting BLSTM is trained separately because it is designed to predict a single value for the entire season, rather than a time series. The outputs for all the time steps of the yield-predicting BLSTM are averaged to obtain a single yield value.
- The phenological state variable BLSTM is trained separately because the fraction of fields in each phenological stage in a county is equivalent to the probability that a particular field in a county is in a particular phenological stage. As a result, a softmax output layer, which forces the outputs to be probabilities that sum to 1, and a cross-entropy cost function must be used.

The physical state variables predicted represent a subset of the variables available in APSIM. They were selected based on both their agronomic relevance and their detectability in the satellite signal. In [28], the LAI, specific leaf area (SLA), surface soil moisture, and green leaf nitrogen biomass are the variables coupled between APSIM and the canopy RT model, indicating that these should be influential on the canopy signal. All of these variables can be calculated from the outputs of the physical state variable BLSTM. The aboveground biomass, the harvested organ biomass (which becomes the crop yield at the end of the season), and the subsurface soil moisture at several levels have also been included for prediction by the BLSTM. The aboveground and harvested organ biomass are included because of their importance in model calibration and because of previous studies showing their retrievability [33,34,36,70,71]. The subsurface soil moisture was included because root-zone soil moisture is critical to accurately model the growth of water-stressed maize and some studies [72,73] have previously shown that maize root-zone soil moisture can be estimated from the water stress-induced change in maize vegetation indices. The recurrent and bidirectional properties of the BLSTM are particularly attractive for root zone soil moisture because the change in vegetation indices has a complex lagging effect [73] with respect to the root zone soil moisture.

In analyzing the results of the physical state variable BLSTM, it is useful to categorize the retrieved variables into separate groups. Several variables outputted by the BLSTM are highly interrelated; for example, an increase in LAI is inherently highly correlated to an increase in total leaf biomass. While the differences in the retrieval performance of variables in the same group can provide an indication of the plant features within a group to which the satellite signal is most sensitive, it is also interesting to look at the relative performance of variables from different groups. To increase the number of groups analyzed, we calculate the SLA and leaf nitrogen percentage (LNP) from the outputs of the BLSTM as

(6) $$SLA=\frac{LAI}{Total\;Leaf\;Biomass},$$

(7) $$LNP=\frac{Leaf\;Nitrogen\;Biomass}{Total\;Leaf\;Biomass}.$$

Neither the SLA nor the LNP are outputted from the BLSTM because it does not make sense to average a ratio which is undefined when some of the crop in the county has either not emerged or has been harvested in the model over a county. However, the performance of the BLSTM in retrieving both the SLA and LNP is very interesting as, unlike the LAI, total leaf biomass, and leaf nitrogen biomass, they are independent of the overall leaf growth and senescence. Therefore, the SLA is calculated from the outputs of the BLSTM when the LAI is greater than 0.1 and the total leaf biomass is greater than 1 kg ha^−1^, while the LNP is calculated when the total leaf biomass is greater than 1 kg ha^−1^ and the leaf nitrogen biomass is greater than 0.001 kg ha^−1^ (0.1% of the total leaf biomass threshold). With these two calculated outputs, the physical state variable BLSTM variables can be organized into the following categories:

- Variables describing leaf growth and senescence (LAI, total leaf biomass, and leaf nitrogen biomass)
- Variables describing major cumulative carbon assimilation (aboveground biomass and harvested organ biomass)
- Specific leaf area
- Leaf nitrogen percentage
- Soil moisture

Variables from these groups are only weakly connected and the number of categories from which retrievals can be performed gives a sense of the number of independent variables that can be predicted by the BLSTM. While some models do interrelate some of these categories, such as the Monteith light use efficiency model which relates the LAI to carbon assimilation and allows the combination of solar radiation and LAI to predict daily carbon assimilation [74], the correlations between these categories are theoretically limited because of the number of external factors affecting the complex biophysical relationships between them. It is important to note that, unlike [74], our retrieval methodology does not use any data except the satellite measurements to predict the state variables. While external data, such as solar radiation or soil data, can allow some of these categories to be more strongly related, using external data would inherently make the retrieval less generalizable as it would assume that the same biophysical relationships hold in all environments.

The phenological stage prediction BLSTM is included in this study because of prior work [75–77] that has shown that maize phenology is detectable from solar reflective satellite measurements. All stages of maize growth from APSIM [43] have been included for prediction by the BLSTM; however, several short stages that usually last only a few days in our APSIM simulations have been merged together due to the eight-day temporal resolution of the satellite measurement time series used. The mapping of the stages is shown in Table S1 in the Supplementary Materials.

A standard k-fold cross-validation data division framework was used to train, validate and test the BLSTMs. Each county was assigned to one of 10 data divisions and for each data division, the BLSTMs were trained with the other nine folds. Out of these nine folds used for training, six are used as the training dataset for gradient descent and three are used as the validation dataset for early stopping. Training is stopped when there is no improvement in the validation dataset over 30 generations. The process was repeated for each fold. By using k-fold cross-validation and assigning different counties to different folds, the prediction performance results presented in this study are derived from BLSTMs that have never been previously exposed to the data being predicted, either for gradient descent or early stopping.

We perform the analysis of the state variable retrievals on the eight-day time scale of the MODIS surface reflectance used for this study. For the state variables, for each time step, we calculated the k-fold cross validated (CV) R^2^ and percentage uncertainty reduced (PRU), which we defined as

(8) $$PRU=100\times \left(1-\frac{CVRMSE_{predicted}}{CVRMSE_{Mean}}\right)[\%],$$

where *CVRMSE*_*Predicted*_ is the k-fold cross validated root mean square error and *CVRMSE*_*Mean*_ is the root mean square error that would have occurred if the mean of the variable for the day of interest across all folds, except the one in which the prediction is being performed, would have been used as the predictor instead of the BLSTM. Both the *CVRMSE*_*Predicted*_ and *CVRMSE*_*Mean*_ are calculated over all counties. Because these performance metrics are calculated independently for each time step, they are measures of the improvement in the retrieval beyond the mean time series of each state variable. This is a stronger test of performance than commonly used in satellite vegetation product validations, where a single R^2^ and RMSE value is calculated for the entire time series [19,78], ignoring the inherent correlation imposed by the typical temporal evolution of the variables [79]. In order to analyze the spatial performance of the retrievals, spatial plots of ESTD for the physical state variable predictions are calculated at particular times within the growing season.

For the phenological state variables, we also analyze the transition dates between the stages predicted by the BLSTM through both k-fold cross validation and comparison with the state-level USDA NASS ground truth data. In order to determine the transition date for both the BLSTM predictions and APSIM simulations, for each transition date, we calculate the cumulative distribution function that indicates which percentage of fields have experienced the transition. This cumulative distribution function is used it to determine the average transition date predicted by either the BLSTM or APSIM.

## 3. APSIM Calibration

### 3.1. Results

**Figure 4.**
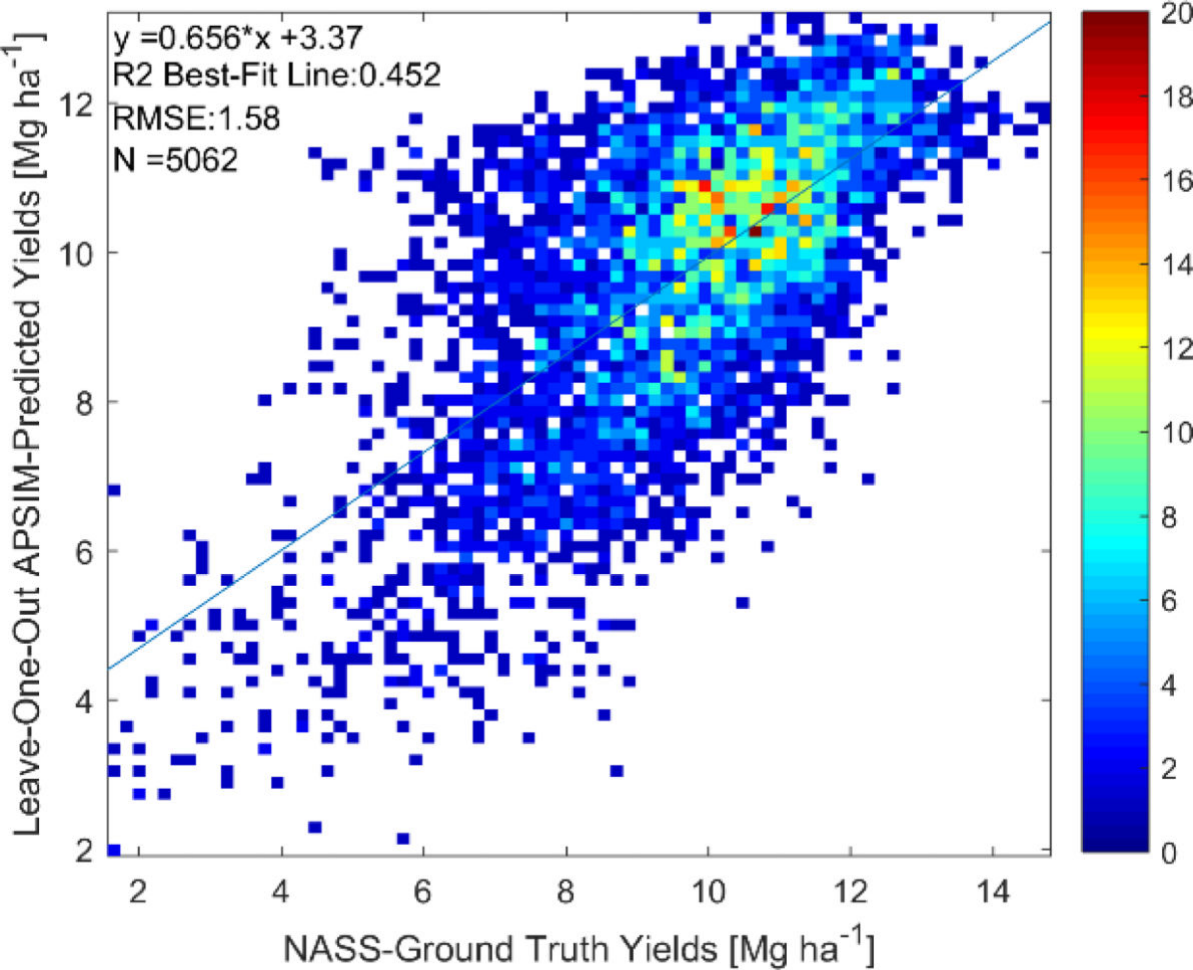
Scatterplot of actual yields versus LOO APSIM-predicted yields for clusterless calibration across entire US Corn Belt. Colorbar represents number of points at a particular pixel in the scatterplot.

As described in Section 2.3.1, calibration of APSIM with respect to county-level yields was performed by both calibrating a constant distribution of agromanagement parameters across the entire US Corn Belt and by calibrating a different distribution for weather-based clusters. In Figure 4 and Table 3, we present the LOO yield and phenological date cross-validation results for the clusterless calibration across the entire US Corn Belt. The results in Figure 4 show a LOO R^2^ value of 0.45 and a LOO RMSE value of 1.58 Mg ha^−1^ for the yield prediction. Furthermore, the phenological stage prediction results in Table 3 comparing to the state-level USDA NASS ground-truth show a LOO R^2^ value of 0.39 and LOO RMSE value of 8.35 days for the prediction of the length of the season. In addition, the LOO R^2^ for the three phenological dates were predicted with values above 0.8.

**Figure 5.**
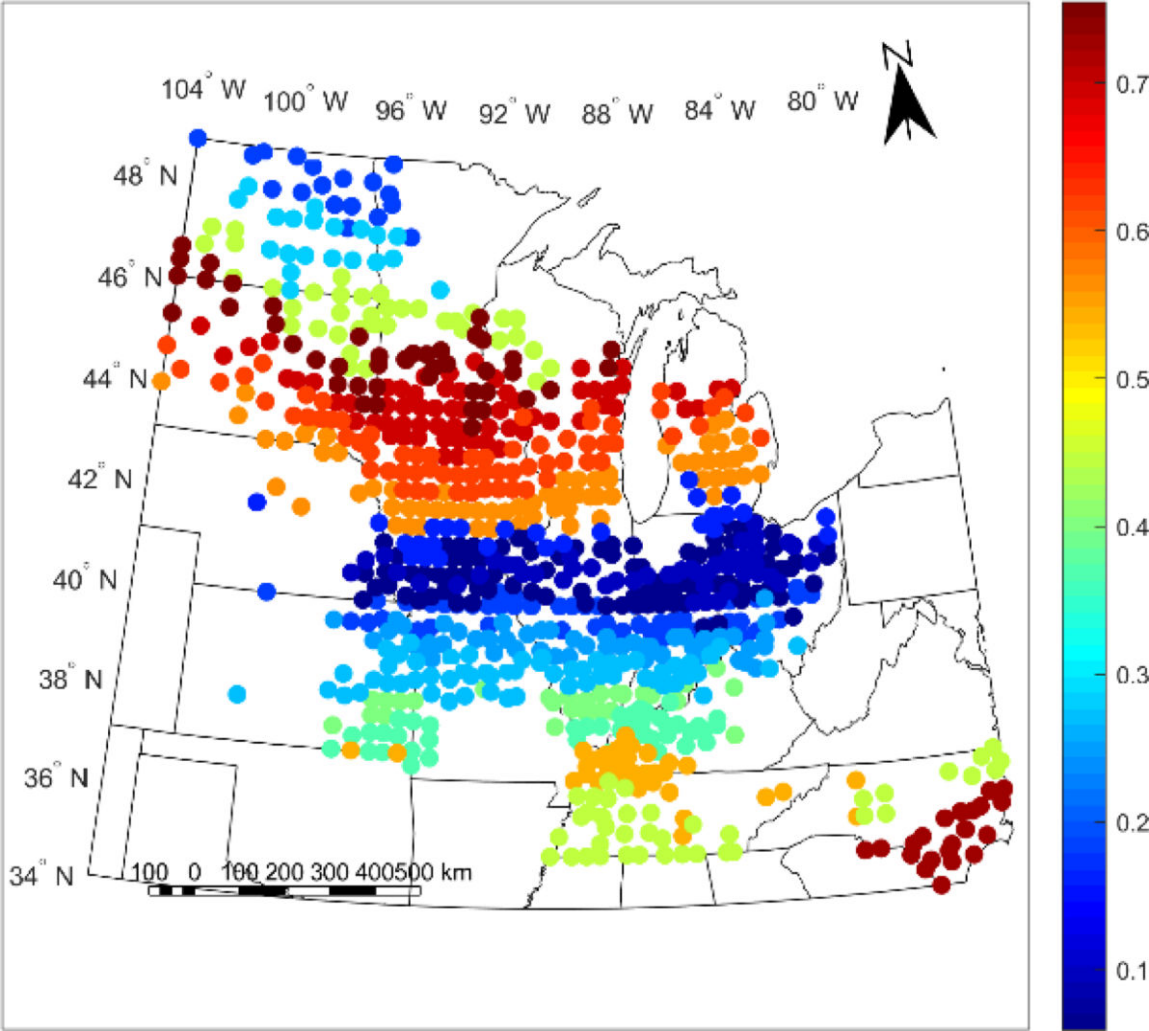
LOO yield prediction R^2^ values by cluster for the 20-cluster weather-based clustering calibration.

**Figure 6.**
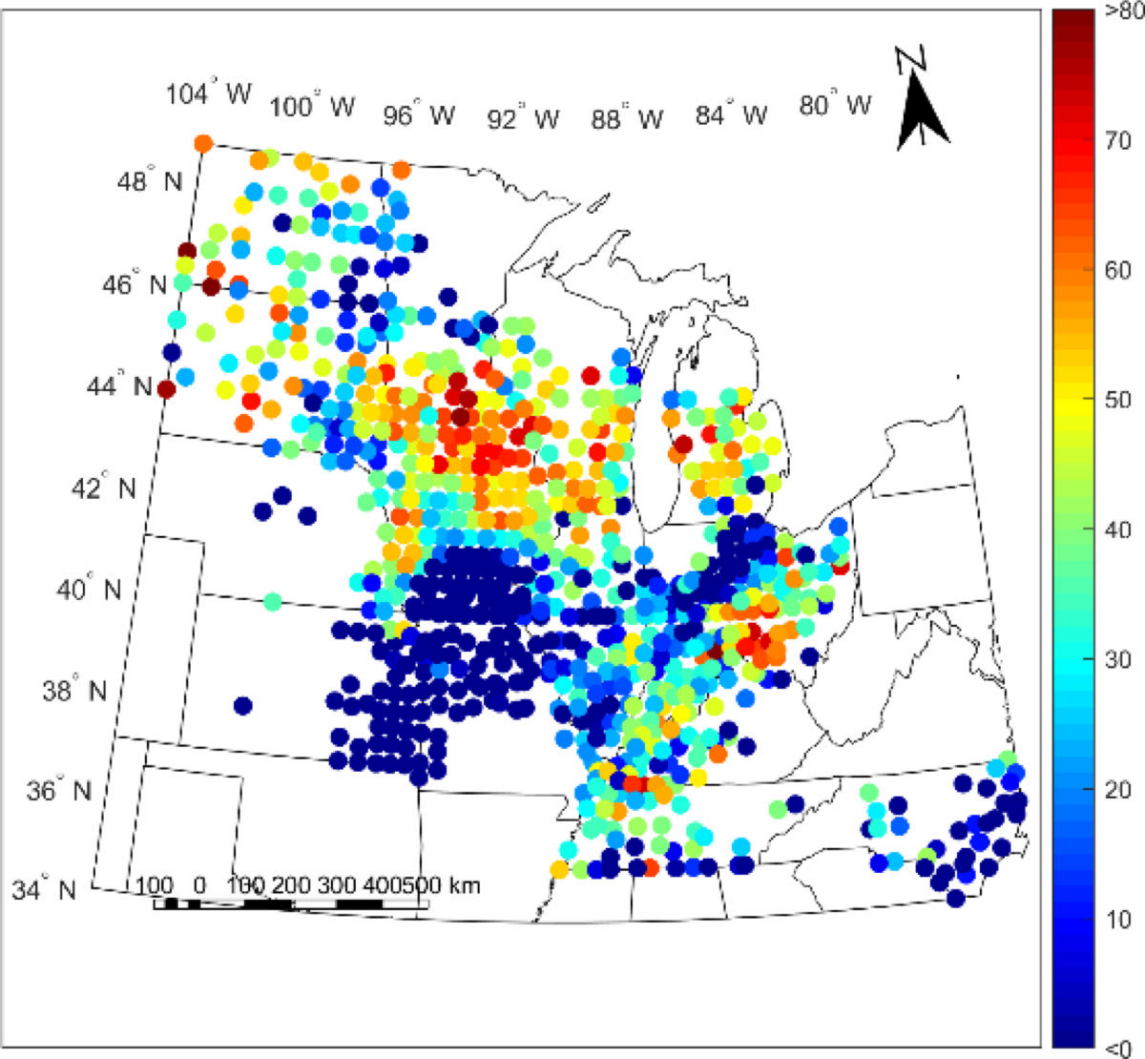
LOO yield prediction ESTD values (%) averaged for each county for the 20-cluster weather-based clustering calibration.

For the weather-cluster-based calibration, we chose to use 20 clusters and in Figures 5 and 6, we present the LOO R^2^ and ESTD for yield prediction for each cluster in this calibration. In Figure 5, each county is assigned the R^2^ value of its corresponding cluster, while in Figure 6, the ESTD value is the value for the county itself over the study period years. Figure 5 is stratified because each cluster is assigned the value of the R^2^ value calculated with all county-years within the cluster; because of this stratification, the clusters used for this study can be seen as each region with a different color in Figure 5. By calculating the R^2^ value for all county-years within the cluster, the overall spatiotemporal performance of the model within the cluster is seen.

As can be seen from the results in Figures 5 and 6, the performance of the calibration between different clusters varies significantly and has a very distinct spatial pattern, with particularly poor model performance in a band from Kansas to northern Indiana. This is seen with LOO R^2^ values below 0.3 and ESTD values below 20% in this region. In contrast, several regions outside this band have higher LOO R^2^ between 0.35 and 0.75 and ESTD values above 40%, indicating strong model performance. Further, while some of the regions with high LOO R^2^ values have low ESTD values, such as North Carolina, this does not necessarily represent that the models in these regions cannot be used, but rather that they are magnitude of the average model bias is greater in these regions, while the variability of the yield is captured correctly.

To verify that the spatial dependence of the model performance seen in Figures 5 and 6 is not solely a result of the clustering chosen, we repeated the calibration with 10 weather-based clusters and 10 purely geographic-based clusters indicating that, broadly, the spatial dependence of the performance is not solely an artifact of the clustering chosen. The LOO R^2^ and ESTD values for these two clusterings are shown in Figures S1 and S2 in the Supplementary Materials. In addition, the ESTD values for the clusterless calibration are shown in Figure S3 and also show remarkable similarities to the ESTD values presented for the different model calibrations in Figure 6, Figures S1 and S2. The common poor model performance in the Kansas to northern Indiana band in all of these ESTD figures shows that there is likely a physical basis for the weak performance across all calibrations.

**Figure 7.**
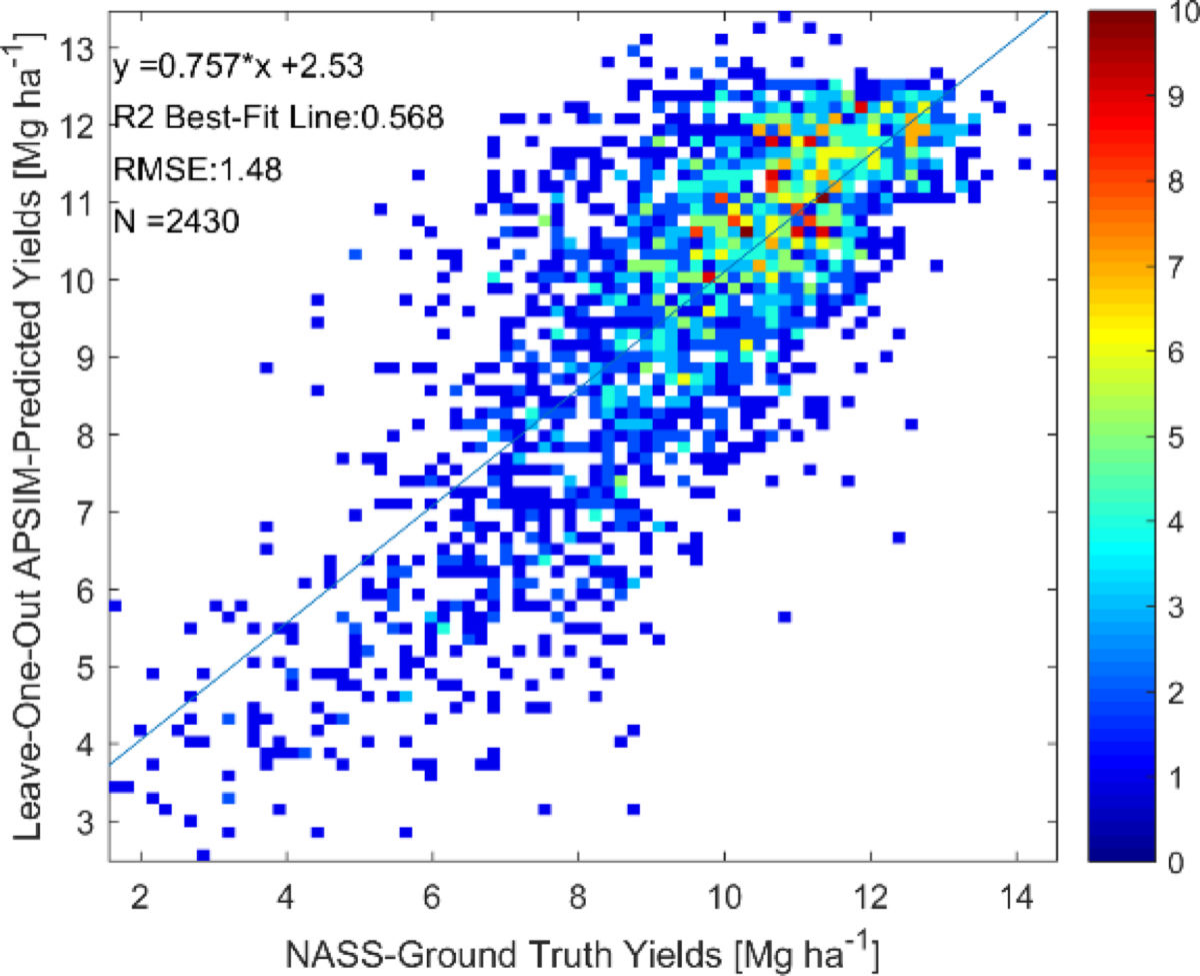
Scatterplot of actual yields versus LOO APSIM-predicted yields for clusters with LOO R^2^ values above 0.40 (using clustering in Figures 5 and 6). Colorbar represents number of points at a particular pixel in the scatterplot.

As a result of the spatial dependence of the model performance, we decided to use high-performing regions to assess the feasibility of retrieving the predicted state variable from collocated satellite measurements using the weather-based-clustering by setting a threshold of only using clusters with overall LOO R^2^ values above 0.40. In Section 4, we refer to these high-performing regions as the “selected weather clusters”. This represents approximately half of the county-years in the dataset. The LOO yield-prediction results for these clusters with this calibration are shown in Figure 7, while the phenological date retrieval results are shown in Table 4. Figure 7 shows that LOO yield performance among the selected clusters is has a LOO R^2^ value of 0.57. Table 4 shows that the length of season is predicted with a LOO R^2^ value of 0.38 and a LOO RMSE of 5.8 days, while phenological dates are predicted with LOO R^2^ values above 0.75.

### 3.2. Discussion

The calibration results show that APSIM can be used to provide realistic simulations of crop growth, especially outside the Kansas to northern Indiana band. The decision to filter the results used for the satellite retrieval analysis based on the quality of model performance was made based on the need for the model to accurately capture the main factors driving crop growth when performing the retrieval feasibility analysis. The LOO R^2^ performance metric serves as a good metric to select regions to assess the satellite retrieval performance because:

- It is high when the yield variability is driven by phenomena that are well-modelled and caused by input factors known to the model, such as intracluster variability in weather and soil, as opposed to factors unknown to the model, such as intracluster variability in genotype, agromanagement practices, pests, weeds, and other factors.
- It is high only when the model generalizes to other counties and years in the region, implying a degree of physicality, due to its cross-validated nature

Filtering was performed with the LOO R^2^ rather than the ESTD to focus on how well the yield variability was captured in each region, rather than the average model bias in the region. This is because we seek regions where the variability in the yield-affecting factors is captured, rather than regions where the error is nominally low.

It is also important that the clusterless calibration over the entire US Corn Belt is used to assess the satellite retrieval feasibility to ensure that biases are not introduced by calibrating separately for each cluster or by excluding certain clusters, testing for greater retrieval algorithm generalizability. While the LOO R^2^ for the clusterless calibration in Figure 4 is lower than that in the selected weather clusters in Figure 7 (0.45 versus 0.57), the decent LOO R^2^ values in Figure 4 show that the calibrated APSIM model robustly models the effect of meteorology and soil variability to predict crop yields, even when it must assume that the same agromanagement parameters are applied over the entire United States. The strong spatial performance of the model under clusterless calibration over the entire United States Corn Belt is also seen by strong spatial performance in the EOF analysis in Figure S4 of the Supplementary Materials; strong spatial performance is critical in generating realistic data for the feasibility analysis.

Although it would be desirable to have better regional crop model performance to conduct this feasibility study, the regional calibration performance attainable by a crop model is inherently limited. As a comparison, [11] found a quadratic relationship between crop model predicted and actual yields with an R^2^ value of 0.59 while using ground weather station data (including solar radiation) and proprietary data on typical variety maturities and planting densities by site from DuPont^®^ and other sources. The availability of this ground-station weather and agromanagement data is likely to have contributed significantly to the performance seen in [11], while limiting the generalizability of the approach to regions where the data is unavailable, which is common even in the United States [56]. For example, the use of gridded weather data in this study, especially the low resolution NASA POWER solar radiation, is likely to have negatively affected the results [56]. Despite the limited data used in this study and despite assuming no variation in the varieties planted across the counties at all, an R^2^ value for the clusterless calibration over the entire US Corn Belt of 0.45 (Figure 4) is obtained to a linear relationship, which is strongly preferable to a quadratic relationship in model validation. Furthermore, the exclusion of poorly-modelled regions and use of cluster-based calibration increases the R^2^ value to 0.57 (Figure 7) in the selected weather clusters. In addition, unlike [11], we also performed calibration against USDA NASS growth season lengths and validated the growth season lengths and three of the APSIM-predicted phenological dates against the state-level USDA NASS ground truth. The validation against the length of season is an important test of the phenological performance of the APSIM calibration as the length of the season was only used as a calibration target. Therefore, due to the nature of LOO cross-validation, the prediction of the length of the season (and the prediction of the yield) has been evaluated on calibrations that have never been exposed to data containing the length of the season in the current county or the current year. The ability of the model to predict the length of the season with LOO RMSE values between 5.8 and 8.4 days and LOO R^2^ values of 0.38 to 0.39 shows that the phenological performance of the model is reasonable, especially considering the eight-day temporal resolution at which the satellite retrieval analysis will be conducted and the uncertainties inherent in the weekly state-level ground truth data. Accurate simulations of the length of the season are dependent on accurate determination of the seed variety distributions from the calibration against USDA state-level crop progress report season lengths and county-level yields. We are unaware of any other studies reporting regional-scale crop growth model performance against USDA state-level crop progress report phenological dates and thus it is likely that additional data, such as the proprietary seed relative maturity data obtained for select sites in [11], is necessary to improve the prediction of the length of the season. However, the accuracy of the data for select sites in [11] is unknown as validation of the phenology is not conducted in [11]. Furthermore, restricting our study to the select sites would limit our study’s geographic extent and generalizability. The phenological date LOO R^2^ values, which are all above 0.75, are significantly higher than those for the length of the season because the planting date percentiles are inputs into the APSIM simulations and are inherently correlated with the phenological dates. Therefore, unlike the length of the season, the APSIM phenological date prediction performances are not independent of the APSIM model inputs and cannot be solely used to assess the phenological performance of the model; however, the decent LOO RMSE between 0.60 and 1.55 weeks provide confidence in the physicality of the simulations with respect to timing.

## 4. Retrieval of Predicted State Variables from Satellite Measurements

### 4.1. Results

**Figure 8.**
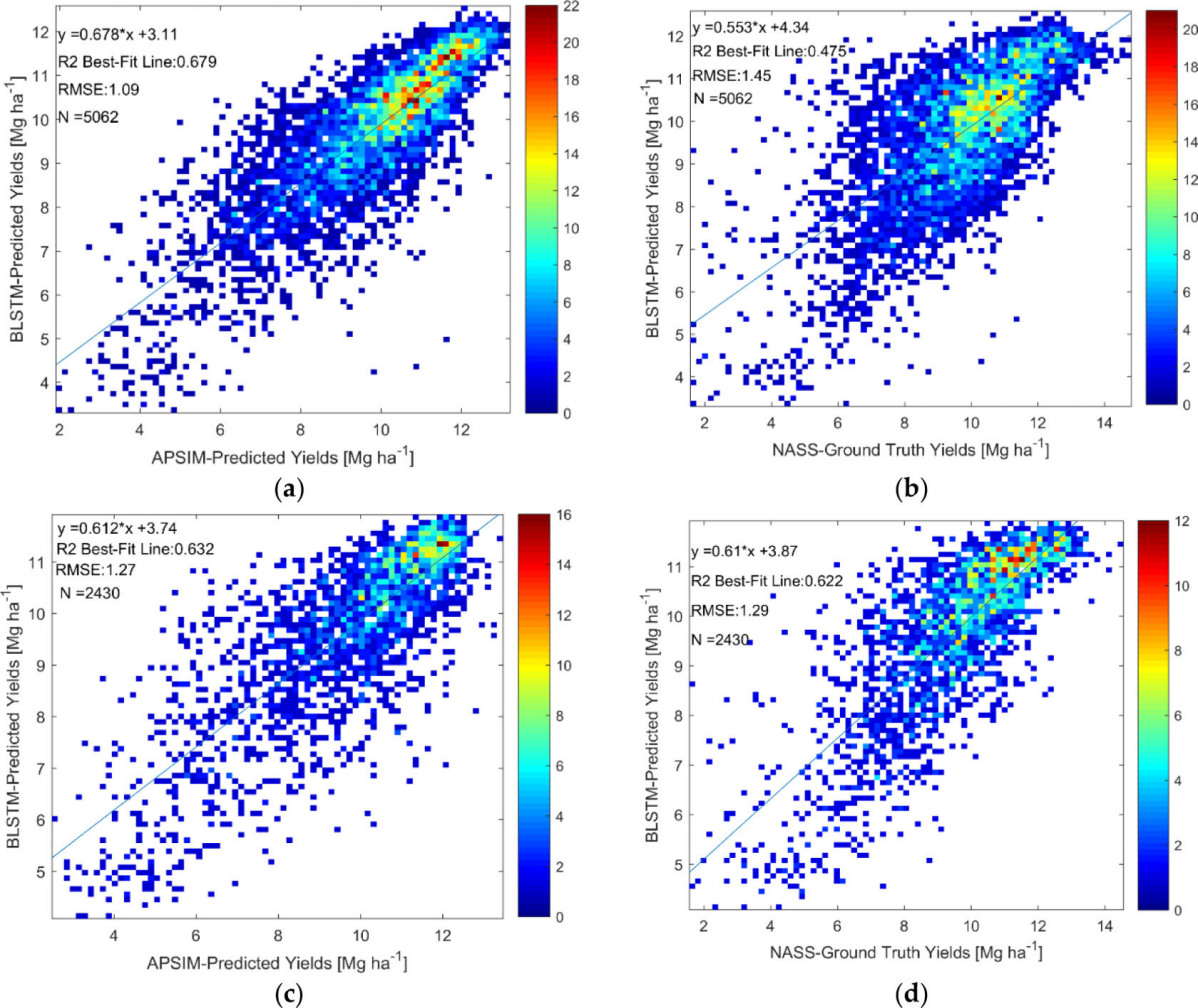
Scatterplots of BLSTM-predicted versus APSIM-predicted (**a**,**c**) and BLSTM-predicted versus NASS ground-truth (**b**,**d**) yields (**a**,**b**) over the entire US Corn Belt using clusterless calibration and (**c**,**d**) in selected weather clusters using the 20-cluster weather-cluster-based calibration. Colorbars represent number of points at a particular pixel in the scatterplot.

We now present the results for the BLSTMs trained to predict the APSIM-simulated agronomic variables. We first present the results from the BLSTMs that predict the APSIM-simulated yields, as these can also be directly compared to the ground-truth county-level USDA NASS survey yields. The performance of the yield-predicting BLSTM with respect to the APSIM-simulated yields and USDA NASS survey yields for both calibrations is shown in Figure 8a-d.

The results in Figure 8 show how well the yield-predicting BLSTMs are able to retrieve both the APSIM-predicted yields, which were used for training and evaluated by k-fold cross-validation, and the actual NASS ground-truth yields, which were never used for training at all. The BLSTM trained on the clusterless calibration data over the entire US Corn Belt can predict the APSIM-simulated yields with a CV R^2^ value of 0.68, while the NASS ground-truth yields are predicted with an R^2^ value of 0.48. The BLSTM trained on the data from the selected weather clusters can predict the APSIM-predicted yields with a CV R^2^ value of 0.63, while the NASS ground-truth yields are predicted with an R^2^ value of 0.62. The results show that while the BLSTMs perform better at retrieving the APSIM-predicted values than actual values, learning to predict APSIM-simulated values does teach the BLSTMs to predict actual values relatively well.

We now present the results of the phenological state variable BLSTMs by evaluating their performance in predicting the transition dates. For these BLSTMs, unlike the yield-predicting BLSTMs, some of the transition dates do not have a ground truth to compare against, necessitating the sole use of k-fold cross-validation to evaluate the performance for these transition dates. The transition date results for both the clusterless calibration across the entire US Corn Belt and the weather-cluster-based calibration in the selected clusters are shown in Table 5. As the USDA ground-truth data is only available at the state level, the BLSTM versus USDA results in Table 5 are based on state-averaged values. In addition, the Supplementary Materials show the CV R^2^ and CV PRU values for the phenological stage membership probabilities themselves, as well as cross-validated confusion matrices, for each calibration, in Figures S5–S8. The kappa coefficient for the stage classifications (based on the confusion matrices in Figures S6 and S8) is 0.82 for the clusterless calibration over the entire US Corn Belt and 0.83 for the weather-cluster-based calibration in the selected weather clusters.

The results in Table 5 show that the phenological state variable BLSTM, trained to predict APSIM-simulated phenological stage membership probabilities, is able to accurately reproduce the APSIM-simulated transition dates and predict the USDA NASS crop progress report median transition dates. Importantly, in both calibration scenarios, the three USDA NASS transition dates considered (emergence, silking, and maturity) are predicted with R^2^ values above 0.75, although there are some biases in some of the predictions, particularly in the maturity date which has RMSEs near 12 days. It is also important to note here that, unlike the APSIM calibration, the BLSTM is not provided with any information about the planting date and, as a result, the results in Table 5 are a valid test of the ability to retrieve the ground-truth phenological dates solely from MODIS measurements. Furthermore, the two transition dates predicted by the BLSTM for which there is no USDA NASS ground-truth data (floral initiation and start grain fill) are predicted with CV R^2^ between 0.69 and 0.83 with respect to the simulated values, which is similar to the range of CV R^2^ (0.55 to 0.85) for the transition dates that do have corresponding ground truth data. As a result, it can be expected that the BLSTM predictions of these two transition dates would have similar performance metrics with respect to ground truth data, had it been available.

**Figure 9.**
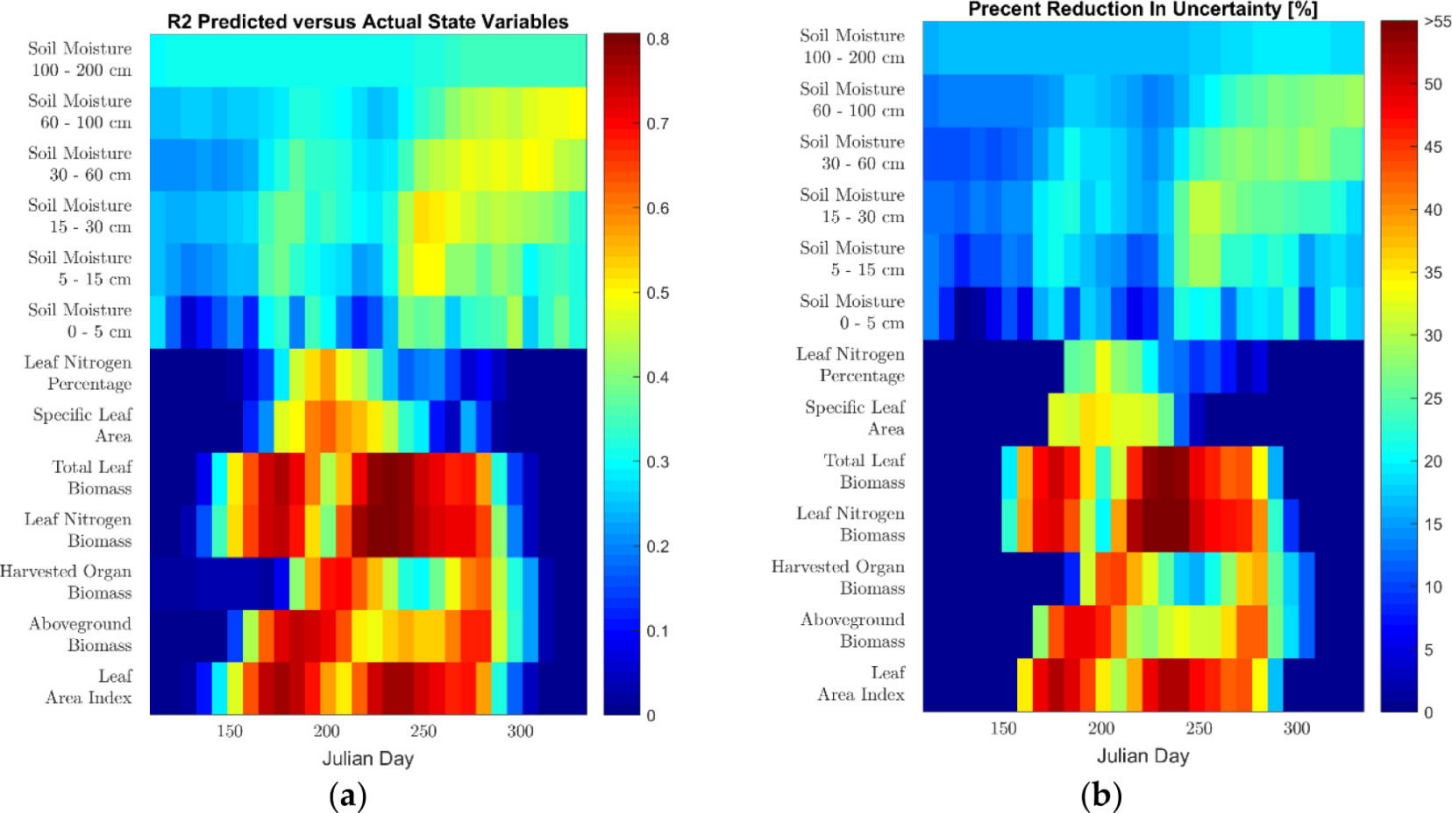
(**a**) CV R^2^ and (**b**) CV PRU physical state variable prediction results for clusterless calibration over entire US Corn Belt.

**Figure 10.**
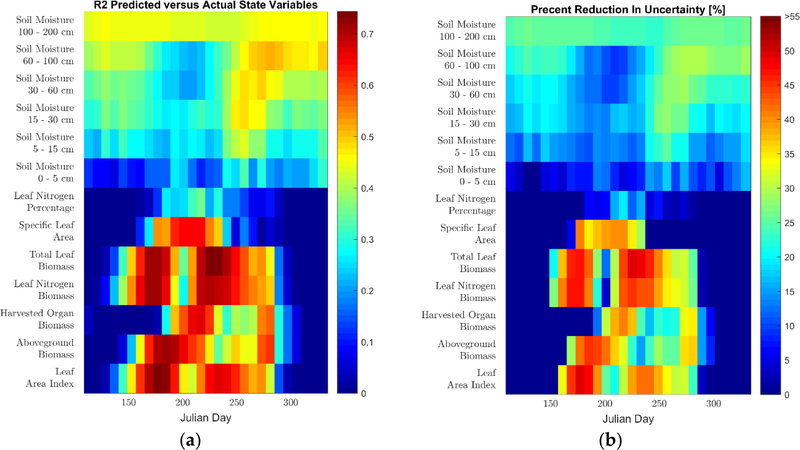
(**a**) CV R^2^ and (**b**) CV PRU physical state variable prediction results in selected weather-based clusters.

Lastly, in Figures 9 and 10, we present the retrieval results for the physical state variables for the two calibration approaches. Figures 9 and 10 show the timestep-by-timestep CV R^2^ and PRU retrieval performance for these predictions. Unlike the yields and phenological state variables, a ground-truth dataset does not exist for these variables at the regional scale and the retrieval performance for these variables is solely assessed through k-fold cross-validation. From the results, one can see that the BLSTMs have a strong predictive ability for the state variables describing the aboveground plant structure, with several having temporal CV R^2^ values between 0.4 and 0.8, along with a 30 to 55% reduction in uncertainty as compared to the CV mean. Specifically, all variables in the leaf growth and senescence category have CV R^2^ values above 0.65 for large portions of the growing season, while the cumulative carbon assimilation category generally ranges in retrieval performance with CV R^2^ values of 0.4 to 0.7 for most of the growing season. Interestingly, especially in terms of PRU for the clusterless calibration, the aboveground biomass is visibly better retrieved than the harvested organ biomass for a large portion of the season, indicating that the satellite signal is possibly more sensitive to biomass than grain yield. Furthermore, the SLA, which provides information about the leaves that is independent of their growth and senescence, is predicted with CV R^2^ values of up to 0.6. The leaf nitrogen percentage, which also provides information about the leaves that is independent of their growth and senescence, is successfully predicted in the clusterless calibration (with CV R^2^ values of up to 0.6); however, it is not well predicted in the selected weather clusters. Lastly, the soil moisture state variables are retrieved with R^2^ values between 0.25 and 0.5 with generally stronger performance later in the season, except the surface layer, which is retrieved significantly less accurately than the other layers. Spatial plots of ESTD for the physical state variable predictions are shown in the Supplementary Materials in Figure S9.

### 4.2. Discussion

Overall, the results presented in Section 4.1 demonstrated the possibility of retrieving several agronomic variables from solar reflective satellite measurements via a new methodology of training agronomic satellite retrieval algorithms solely with collocated crop growth model simulations. Because our methodology only requires collocated crop growth model simulations, rather than collocated measurements, we are able to explore the performance of our method for both variables that have ground-truth measurements and those that do not.

First, we discuss the performance of the BLSTMs which had ground-truth data for validation. This validation of these BLSTMs is very important to show that although the BLSTMs only see crop growth model simulated values in training, they are able to predict actual values measured on the ground. The NASS ground-truth yields are predicted with an R^2^ value between 0.475 and 0.62, depending on the calibration used. As a comparison, a phenology-based regression approach produced a cross-validated R^2^ value of 0.59 for county-level maize yield prediction [80], a neural network approach to county-yield prediction provided an R^2^ of 0.78 in [81], and an approach based on CM-Reg provided an R^2^ of 0.74 in [34]. While our results are not as good in terms of yield prediction as the best results in the literature, this is entirely to be expected with our method, as we only trained on APSIM-simulated yields that themselves only had LOO R^2^ between 0.45 and 0.57 with the actual NASS ground-truth county-level yields. The fact that we were able to predict actual yields from the satellite with this strong of a performance despite training only on the APSIM-simulated yields provides strong validation of our methodology, as our goal is not to create another yield prediction method, but to be able to use crop growth model simulations to learn to retrieve variables for which there is no ground truth data for the satellite measurements. Furthermore, the actual state-level USDA NASS crop progress reports transition dates are predicted with high R^2^ (above 0.75), as seen in Table 5. Particularly notable is the USDA NASS state-level median silking date for the clusterless calibration across the entire US Corn Belt, which is predicted with an R^2^ value of 0.85 and a RMSE of 4.2 days. As a comparison, a very recent paper [77] expanding on previous work with the shape-fitting method [36,76], predicted the USDA NASS silking date with an RMSE of 4.3–4.5 days and an R^2^ value of 0.85 to 0.88 across the US Corn Belt. To our knowledge, [77] represents the current state-of-the-art in regional satellite maize phenology retrieval and it is impressive that our method, which is trained only on APSIM simulations, can match its performance for the silking date, which is critical in agronomy and field-scale crop model simulations [82]. The two other USDA NASS transition dates are predicted in [77] with very similar R^2^ values to our values in Table 5; however, our method produces higher RMSE values. The higher RMSE for the other USDA NASS transition dates are, however, to be expected, as the APSIM-predictions themselves in Tables 3 and 4 have similar RMSE values as compared to the USDA NASS transition dates. In addition, while discussing the performance of the variables which had ground truth data, we wish to note the differences between the performance results obtained by k-fold cross-validation comparison to APSIM-simulated variables (the sole method available to evaluate the performance of the variables that do not have ground-truth data) and those obtained by comparison to ground-truth data. For the crop yield retrievals in Figure 8, the R^2^ value decreases from 0.68 to 0.475 when comparing to ground-truth yields instead of APSIM-simulated yields when looking at the clusterless calibration across the entire US Corn Belt, while the R^2^ value only decreases from 0.63 to 0.62 when looking at performances of the selected weather clusters. These results indicate that, although k-fold cross-validation with respect to APSIM-simulated data can overestimate the retrieval performance with respect to ground truth data, the magnitude of the overestimation varies and decreases when the APSIM model performance is stronger. The silking and maturity date retrievals in Table 5 show much smaller differences in terms of R^2^ values between the comparison to APSIM-simulated dates and ground-truth values than the yield retrievals and surprisingly the emergence date retrievals have higher R^2^ values toward the ground-truth data then toward the APSIM-simulated values, indicating that in some cases the BLSTM can use the MODIS data to learn to remove the noise from the APSIM simulations and retrieve the actual values better than the APSIM data on which it was trained.

We now discuss the performance of our methodology with respect to the physical state variable BLSTM, whose variables did not have ground truth data and were thus evaluated solely via k-fold cross-validation. The results for both calibrations showed strong performance in retrieving information from three categories of variables: leaf growth and senescence, cumulative carbon assimilation, and SLA. All three of these categories are retrieved with CV R^2^ between 0.4 and 0.8 and CV PRU values between 30% to 55% for significant portions of the season for both calibrations, although the SLA is retrieved for a shorter portion of the season than the others. This ability of the BLSTMs to reproduce these APSIM-simulated variables indicates that it is likely that this methodology will be able to accurately predict actual physical state variable time series, particularly if this method is reproduced with field-scale crop simulations and collocated satellite imagery with data from cooperating farmers. The retrieval of SLA by our algorithm is particularly interesting, as it is rarely retrieved from space-borne instruments; a review [31] of maize remote sensing found no studies retrieving SLA and we are only aware of two [83,84] for any types of vegetation at all. Lastly, the soil moisture retrieval results show some promise for this methodology with CV R^2^ values between 0.25 and 0.5 and CV PRU values of up to 30%. This is particularly true if the methodology is reproduced at the field scale, where the modeling of soil water transport is expected to be significantly more accurate [21]. Interestingly, the surface layer, which is the only one that can be directly observed by the satellite, is retrieved with the lowest quality. This poor performance at the surface may be explained due to the attenuation of the surface soil signal by the plant canopy as the canopy closes; in contrast, the soil moisture in the deeper layers is likely being predicted by the BLSTM due to its detection of water stress in the leaf reflectance and its use of its bidirectional structure to learn the appropriate lag [73] between soil moisture changes and plant water status.

Furthermore, except for the leaf nitrogen percentage, the results for the retrieval of the physical state variables were quite similar using the two different calibrations, providing further support for the feasibility of our methodology. The retrieval results in the selected weather clusters do appear to be slightly inferior to those using the clusterless calibration over the entire US Corn Belt; however, the differences for all the variables except the leaf nitrogen percentage are not too large and some differences are expected due to strength and weaknesses of each calibration approach. The large difference in the retrieval performance of the leaf nitrogen percentage, which was retrieved quite well in the clusterless calibration and quite poorly in the selected weather clusters, may indicate that our APSIM simulations may insufficiently model the effects of nitrogen stress when looking at a subnational scale. The availability of actual fertilization rates if this method is reproduced with field-scale data has the potential to resolve this issue and potentially allow the leaf nitrogen percentage to be retrieved. Overall, beyond the leaf nitrogen percentage, the strong feasibilities shown with both approaches gives us confidence in our results.

The verification of the ability to retrieve the county-level state variables in the results implies that there is a strong possibility that if a large dataset of geolocated, field-scale agromanagement records were to be collected, the method could be reproduced to predict the field-scale state variables by training with field-scale agromanagement data. Although it is very difficult for researchers to gain farmer’s trust to obtain agromanagement data for a large number of fields [85] to train an operational version of this retrieval methodology, it is possible with a concerted effort by group of researchers working extensively and collaboratively with farmers. For example, under promises of strict data secrecy, studies in the literature have collected thousands of field-years of data via surveying efforts [86] that is similar to the data that would be required for training these field-scale BLSTMs.

The growth of precision agriculture and the automated data collection provided by its infrastructure [5,85] may allow for the efficient collection of data to train these field-scale algorithms. It is likely that high-resolution satellite imagery fused [87] with moderate resolution satellite images (such as 10- to 20-m Sentinal-2 data and 30-m Landsat data fused with 250-m and 500-m MODIS data) will be better suited for training these field-scale BLSTMs than using only MODIS data because there frequently exists significant within-field growth variability caused by the inhomogeneity of soil and management practices in the field [5,88,89]. Furthermore, surveyed field plots may be smaller than the 500-m MODIS pixels [90]. The main disadvantage of high resolution data is its high revisit time; however, data fusion, using algorithms such as the Spatial and Temporal Adaptive Reflectance Fusion Model (STARFM) [91], allows for this shortcoming to be mitigated, especially with the addition of newer satellite systems that provide the fusion algorithms with more observations, such as the two Sentinal-2 satellites launched in 2015 and 2017 that provide a 5-day revisit time (as opposed to the 16-day revisit time traditionally provided by Landsat) [87]. Future work is needed to assess the effect that using fused high-resolution/moderate resolution data will have on this methodology, particularly because the quality of the data fusion is strongly dependent on the inhomogeneity of the field [91] (i.e., the performance of STARFM should be much better on a homogenous field as compared to an inhomogeneous field). Furthermore, commercial platforms, such as the Planet Labs satellites that have daily revisit time with a constellation of satellites, provide an additional option for addressing the issue of temporal resolution. Overall, the development [87] of fusion products with publicly available [92] data from new, lower revisit time high resolution satellite systems, such as Sentinal-2, and the potential availability of daily data from commercial sources gives us confidence that this algorithm can be trained at field scale in the future.

Once an operational version of the methodology is trained with field-scale data, it can provide information on the in-season state variables of maize growth on a global scale. In the operational phase, there is also an additional issue of identifying the maize pixels on which this algorithm should run using a crop classification product, which is more difficult in real-time and outside the United States because few crop classification products have as high quality as the retrospective USDA NASS Cropland Data Layer [53] used in this study, produced annually for the Contiguous United States after the end of the growing season. However, crop classification from satellite remote sensing is a very active, rapidly progressing area of research [93–99] for data both inside and outside the United States, at high resolution and at moderate resolution, and in real-time during the growing season and retrospectively; therefore, the availability of crop classification products should not present a great hurdle to adaption of this approach globally.

## 5. Conclusions

In this study, we used regional crop growth modeling to assess the feasibility of using collocated crop growth model simulations and satellite measurements to train an empirical satellite agronomic variable retrieval algorithm with bidirectional long short-term memory networks. Confidence was built in the methodology by verifying that an algorithm trained solely with collocated crop growth model simulations (without any ground-truth data) could accurately predict ground-truth values for the agronomic variables for which it was available (the yields and phenological transition dates). We then used k-fold cross-validation to explore the retrieval of variables that did not have ground-truth data. In these analyses, we showed that three categories of physical state variables that lack regional-scale ground-truth time-series data (leaf growth and senescence, cumulative carbon assimilation, and specific leaf area) can be retrieved from the remote sensing measurements with cross-validated R^2^ values ranging from 0.4 to 0.8 for significant portions of the season. The results also showed that it is potentially possible to retrieve some amount of information about further variables, such as the soil moisture. The methodology proposed in this study provides a realistic, consistent methodology that can be used by future survey efforts of farmer agromanagement data to train systems that are able to retrieve in-season crop growth variables in the face of significant G × E × M variability.

As has been noted [31], the generalizability of retrieval algorithms to new locations and environments is the most important factor limiting the use of remote sensing for crop growth modeling. In this proposed approach, the issue of generalizability can be addressed by drastically increasing the amount of data used to train the retrieval algorithm because one only needs the field agromanagement data, rather than physical in-season measurements of the state variables. Specifically, by using the field agromanagement data to perform crop growth model simulations at the field sites, one can replace actual in-season measurements with simulated in-season state variables in training a satellite retrieval algorithm. This provides the promise of training a strong retrieval algorithm that has constant internal parameters across all locations, regions, and environments if sufficient geolocated agromanagement data is obtained. As stated previously, in light of the expense of field campaigns, this would be potentially a very attractive alternate approach to learning about crop growth on a global scale.

Future work can be conducted to explore the feasibility of incorporating synthetic aperture radar (SAR) data into this methodology and expanding to other crops. Satellite-based SAR data, such as the high resolution SAR data from the Sentinal-1A satellite launched in 2014, can be used to improve retrievals of maize leaf area index [100], biomass [100], crop water requirements [101], and soil moisture [102], indicating that incorporating it into this methodology may allow for further improvement of the retrievals. Morphological-based SAR scattering models can also be used to potentially retrieve other parameters, such as the crop height [103]. Furthermore, this methodology can be expanded to other crops as maize is far from unique in being amiable to satellite remote sensing; for example, a recent study [37] showed that county-level yields for 9 out of 10 major US crop types are significantly correlated to Moderate Resolution Imaging Spectroradiometer (MODIS) vegetation indices, indicating the potential of applying this method to these crops, although the performance will also strongly depend on the quality of the crop growth models for these crops.

## Supplementary Material

S001

## Figures and Tables

**Table 1.** Input variables used for APSIM simulations.

Module	Variable	Source
Maize	Planting Density	Calibrated
	Planting Date	USDA NASS Crop Progress Reports/Calibrated
	Seed Variety	Calibrated
	Nitrogen Fertilizer Applied	Calibrated
	Irrigation Applied (0 if rainfed)	Assumed zero by using only rainfed counties

Weather	Daily maximum temperature	PRISM (tmax)
	Daily minimum temperature	PRISM (tmin)
	Daily precipitation	PRISM (ppt)
	Daily solar radiation	NASA POWER (srad)

Soil	Drained upper limit	POLARIS (theta_33)
	Drained lower limit	POLARIS (theta_1500)
	Bulk density	POLARIS (bd)
	Soil pH	POLARIS (ph)
	Organic matter	POLARIS (om)
	Clay content	POLARIS (clay)
	Saturated water content	POLARIS (theta_s)
	Air dry water content	POLARIS (theta_r)
	Crop lower limit	Set equal to drained lower limit according to [21]
	Maize soil/root water extraction coefficient	Default profile from [21]
	Root penetration parameter	Default profile from [21]
	Soil evaporation coefficients (U and CONA)	Estimated from percent clay following [21]
	Soil water conductivity (SWCON)	Estimated from saturated water content following [21]
	(diffus_const and diffuse_slope)	Default values from [21]
	Soil albedo	Default value from [21]
	Cn2bare	Default APSIM value
	Organic carbon	Estimated from organic matter following [21]
	Organic carbon partitioning coefficients (FBIOM and FINERT)	Default values from [21]
	Initial nitrogen profile	Default APSIM profile

**Table 2.** Possible parameter values considered in APSIM optimizations.

Parameters	Values	Source
Planting Density	6, 7.5, 9 plants m^−2^	[59]
Seed Brand	A, B	APSIM Default Cultivars
Seed Relative Maturity	80, 90, 100, 105, 110, 115, 120, 130 days	APSIM Default Cultivars
Nitrogen Applied	200, 300 kg ha^−1^	[33]
Planting Date	25th, 50th, and 75th percentile of planting progress for state in year in which simulation is performed	USDA NASS Crop Progress Reports

**Table 3.** State-level performance of clusterless calibration in predicting phenological stage transition dates as compared to ground-truth USDA NASS crop progress report data over entire US Corn Belt.

Transition	LOO RMSE (days)	LOO R^2^
Emergence	6.67	0.91
Silking	4.77	0.88
Maturity	10.86	0.81
Length of Season	8.35	0.39

**Table 4.** State-level performance of the 20-weather-cluster-based calibration in predicting phenological stage transition dates as compared to ground-truth USDA NASS crop progress report data for clusters with LOO R^2^ values above 0.40.

Transition	LOO RMSE (days)	LOO R^2^
Emergence	7.56	0.85
Silking	4.30	0.80
Maturity	9.92	0.76
Length of Season	5.80	0.38

**Table 5.** BLSTM performance in retrieving phenological stage transition dates.

	Clusterless Calibration over Entire US Corn Belt				Weather-Cluster-Based Calibration in Selected Weather Clusters			
	
	BLSTM vs. APSIM		BLSTM vs. USDA		BLSTM vs. APSIM		BLSTM vs. USDA	

Transition	CV RMSE (days)	CV R^2^	RMSE (days)	R^2^	CV RMSE (days)	CV R^2^	RMSE (days)	R^2^
Emergence	6.88	0.63	8.42	0.86	9.29	0.55	11.56	0.79
Floral Initiation	4.71	0.76	-	-	5.30	0.69	-	-
Silking	4.97	0.82	4.19	0.85	5.09	0.75	4.84	0.78
Start Grain Fill	5.27	0.83	-	-	5.38	0.77	-	-
Maturity	6.46	0.85	11.46	0.83	6.78	0.75	12.36	0.75

## References

[1] Levitan N; Gross B Assessment of the Information Content in Solar Reflective Satellite Measurements with Respect to Crop Growth Model State Variables. In Proceedings of the 14th International Conference on Precision Agriculture, Montreal, QC, Canada, 24–27 June 2018; International Society of Precision Agriculture: Monticello, IL, USA, 2018; pp. 1–12.

[2] Rosenzweig C; Elliott J; Deryng D; Ruane AC; Müller C; Arneth A; Boote KJ; Folberth C; Glotter M; Khabarov N; Assessing agricultural risks of climate change in the 21st century in a global gridded crop model intercomparison. Proc. Natl. Acad. Sci. USA 2014, 111, 3268–3273. 24344314 10.1073/pnas.1222463110 PMC3948251

[3] Lecerf R; Ceglar A; López-Lozano R; Van Der Velde M; Baruth B Assessing the information in crop model and meteorological indicators to forecast crop yield over Europe. Agric. Syst 2018, 168, 191–202.

[4] Woodard JD Integrating high resolution soil data into federal crop insurance policy: Implications for policy and conservation. Environ. Sci. Policy 2016, 66, 93–100.

[5] Oliver YM; Robertson MJ; Wong MTF Integrating farmer knowledge, precision agriculture tools, and crop simulation modelling to evaluate management options for poor-performing patches in cropping fields. *Eur. J. Agron*. 2010, 32, 40–50.

[6] Grassini P; Wolf J; Tittonell P; Hochman Z Yield gap analysis with local to global relevance—A review. *Field Crop. Res*. 2013, 143, 4–17.

[7] Huffman T; Qian B; De Jong R; Liu J; Wang H; McConkey B; Brierley T; Yang J; Jong D Upscaling modelled crop yields to regional scale: A case study using DSSAT for spring wheat on the Canadian prairies. Can. J. Soil Sci 2015, 95, 49–61.

[8] van Bussel LGJ; Grassini P; Van Wart J; Wolf J; Claessens L; Yang H; Boogaard H; de Groot H; Saito K; Cassman KG; From field to atlas: Upscaling of location-specific yield gap estimates. *Field Crop. Res*. 2015, 177, 98–108.

[9] Deryng D; Sacks WJ; Barford CC; Ramankutty N Simulating the effects of climate and agricultural management practices on global crop yield. Glob. Biogeochem. Cycles 2011, 25.

[10] Müller C; Elliott J; Chryssanthacopoulos J; Arneth A; Balkovic J; Ciais P; Deryng D; Folberth C; Glotter M; Hoek S; Global gridded crop model evaluation: Benchmarking, skills, deficiencies and implications. *Geosci. Model Dev*. 2017, 10, 1403–1422.

[11] Morell FJ; Yang HS; Cassman KG; Van Wart J; Elmore RW; Licht M; Coulter JA; Ciampitti IA; Pittelkow CM; Brouder SM; Can crop simulation models be used to predict local to regional maize yields and total production in the U.S. Corn Belt? *Field Crop. Res*. 2016, 192, 1–12.

[12] Xiong W; Skalský R; Porter CH; Balkovicˇ J; Jones JW; Yang D Calibration-induced uncertainty of the EPIC model to estimate climate change impact on global maize yield. *J. Adv. Model. Earth Syst*. 2016, 8, 1358–1375.

[13] Teixeira EI; Zhao G; de Ruiter J; Brown H; Ausseil A-G; Meenken E; Ewert F The interactions between genotype, management and environment in regional crop modelling. *Eur. J. Agron*. 2017, 88, 106–115.

[14] Ewert F; van Ittersum MK; Heckelei T; Therond O; Bezlepkina I; Andersen E Scale changes and model linking methods for integrated assessment of agri-environmental systems. *Agric. Ecosyst. Environ*. 2011, 142, 6–17.

[15] Angulo C; Rötter R; Lock R; Enders A; Fronzek S; Ewert F Implication of crop model calibration strategies for assessing regional impacts of climate change in Europe. *Agric. For. Meteorol*. 2013, 170, 32–46.

[16] Tatsumi K Effects of automatic multi-objective optimization of crop models on corn yield reproducibility in the U.S.A. Ecol. Model 2016, 322, 124–137.

[17] Weiss M; Troufleau D; Baret F; Chauki H; Prévot L; Olioso A; Bruguier N; Brisson N Coupling canopy functioning and radiative transfer models for remote sensing data assimilation. *Agric. For. Meteorol*. 2001, 108, 113–128.

[18] Jacquemoud S; Verhoef W; Baret F; Bacour C; Zarco-Tejada PJ; Asner GP; François C PROSPECT + SAIL models: A review of use for vegetation characterization. *Remote Sens. Environ*. 2009, 113, S56–S66.

[19] Houborg R; McCabe M; Cescatti A; Gao F; Schull M; Gitelson A Joint leaf chlorophyll content and leaf area index retrieval from Landsat data using a regularized model inversion system (REGFLEC). *Remote Sens. Environ*. 2015, 159, 203–221.

[20] Yu K; Lenz-Wiedemann V; Chen X; Bareth G Estimating leaf chlorophyll of barley at different growth stages using spectral indices to reduce soil background and canopy structure effects. *ISPRS J. Photogramm. Remote Sens*. 2014, 97, 58–77.

[21] Archontoulis SV; Miguez FE; Moore KJ Evaluating APSIM maize, soil water, soil nitrogen, manure, and soil temperature modules in the Midwestern United States. *Agron. J*. 2014, 106, 1025–1040.

[22] Kersebaum KC; Boote KJ; Jorgenson JS; Nendel C; Bindi M; Frühauf C; Gaiser T; Hoogenboom G; Kollas C; Olesen JE; Analysis and classification of data sets for calibration and validation of agro-ecosystem models. *Environ. Model. Softw*. 2015, 72, 402–417.

[23] Hunt LA; Boote KJ Data for Model Operation, Calibration, and Evaluation; Springer: Dordrecht, The Netherlands, 1998; pp. 9–39.

[24] Combal B; Baret F; Weiss M; Trubuil A; Macé D; Pragnère A; Myneni R; Knyazikhin Y; Wang L Retrieval of canopy biophysical variables from bidirectional reflectance: Using prior information to solve the ill-posed inverse problem. *Remote Sens. Environ*. 2003, 84, 1–15.

[25] Baret F; Houles V; Guerif M Quantification of plant stress using remote sensing observations and crop models: The case of nitrogen management. *J. Exp. Bot*. 2006, 58, 869–880. [PubMed] 10.1093/jxb/erl231 17220515

[26] Duan S-B; Li Z-L; Wu H; Tang B-H; Ma L; Zhao E; Li C Inversion of the PROSAIL model to estimate leaf area index of maize, potato, and sunflower fields from unmanned aerial vehicle hyperspectral data. *Int. J. Appl. Earth Obs. Geoinf*. 2014, 26, 12–20.

[27] Zhang L; Guo CL; Zhao LY; Zhu Y; Cao WX; Tian YC; Cheng T; Wang X Estimating wheat yield by integrating the WheatGrow and PROSAIL models. *Field Crop. Res*. 2016, 192, 55–66.

[28] Machwitz M; Giustarini L; Bossung C; Frantz D; Schlerf M; Lilienthal H; Wandera L; Matgen P; Hoffmann L; Udelhoven T Enhanced biomass prediction by assimilating satellite data into a crop growth model. *Environ. Model. Softw*. 2014, 62, 437–453.

[29] Thorp KR; Wang G; West AL; Moran MS; Bronson KF; White JW; Mon J Estimating crop biophysical properties from remote sensing data by inverting linked radiative transfer and ecophysiological models. *Remote Sens. Environ*. 2012, 124, 224–233.

[30] Schlemmer M; Gitelson A; Schepers J; Ferguson R; Peng Y; Shanahan J; Rundquist D Remote estimation of nitrogen and chlorophyll contents in maize at leaf and canopy levels. *Int. J. Appl. Earth Obs. Geoinf*. 2013, 25, 47–54.

[31] Corti M; Cavalli D; Cabassi G; Marino Gallina P; Bechini L Does remote and proximal optical sensing successfully estimate maize variables? A review. *Eur. J. Agron*. 2018, 99, 37–50.

[32] Sibley AM; Grassini P; Thomas NE; Cassman KG; Lobell DB Testing Remote Sensing Approaches for Assessing Yield Variability among Maize Fields. *Agron. J*. 2014, 106, 24.

[33] Lobell DB; Thau D; Seifert C; Engle E; Little B A scalable satellite-based crop yield mapper. *Remote Sens. Environ*. 2015, 164, 324–333.

[34] Jin Z; Azzari G; Lobell DB Improving the accuracy of satellite-based high-resolution yield estimation: A test of multiple scalable approaches. Agric. For. Meteorol 2017, 247, 207–220.

[35] Clevers JGP A simplified approach for yield prediction of sugar beet based on optical remote sensing data. *Remote Sens. Environ*. 1997, 61, 221–228.

[36] Sakamoto T; Gitelson AA; Arkebauer TJ MODIS-based corn grain yield estimation model incorporating crop phenology information. *Remote Sens. Environ*. 2013, 131, 215–231.

[37] Johnson DM A comprehensive assessment of the correlations between field crop yields and commonly used MODIS products. *Int. J. Appl. Earth Obs. Geoinf*. 2016, 52, 65–81.

[38] Soufizadeh S; Munaro E; McLean G; Massignam A; van Oosterom EJ; Chapman SC; Messina C; Cooper M; Hammer GL Modelling the nitrogen dynamics of maize crops—Enhancing the APSIM maize model. *Eur. J. Agron*. 2018, 100, 118–131.

[39] Heng LK; Hsiao T; Evett S; Howell T; Steduto P Validating the FAO AquaCrop Model for Irrigated and Water Deficient Field Maize. *Agron. J*. 2009, 101, 488.

[40] Yang H; Dobermann A; Lindquist J; Walters D; Arkebauer T; Cassman K Hybrid-maize—A maize simulation model that combines two crop modeling approaches. *Field Crop. Res*. 2004, 87, 131–154.

[41] Ben Nouna B; Katerji N; Mastrorilli M Using the CERES-Maize model in a semi-arid Mediterranean environment. Evaluation of model performance. Eur. J. Agron 2000, 13, 309–322.

[42] Keating B; Carberry P; Hammer G; Probert M; Robertson M; Holzworth D; Huth N; Hargreaves JN; Meinke H; Hochman Z; An overview of APSIM, a model designed for farming systems simulation. *Eur. J. Agron*. 2003, 18, 267–288.

[43] Brown HE; Teixeira EI; Huth NI; Holzworth DP The APSIM Maize Model; APSIM Initiative: Toowoomba, Australia, 2014.

[44] Wang E; Robertson MJ; Hammer GL; Carberry PS; Holzworth D; Meinke H; Chapman SC; Hargreaves JNG; Huth NI; McLean G Development of a generic crop model template in the cropping system model APSIM. *Eur. J. Agron*. 2002, 18, 121–140.

[45] Schauberger B; Archontoulis S; Arneth A; Balkovic J; Ciais P; Deryng D; Elliott J; Folberth C; Khabarov N; Müller C; Consistent negative response of US crops to high temperatures in observations and crop models. *Nat. Commun*. 2017, 8, 13931. [PubMed] 28102202 10.1038/ncomms13931 PMC5253679

[46] Liu Z; Yang X; Lin X; Hubbard KG; Lv S; Wang J Narrowing the Agronomic Yield Gaps of Maize by Improved Soil, Cultivar, and Agricultural Management Practices in Different Climate Zones of Northeast China. Earth Interact. 2016, 20.

[47] Chaney NW; Wood EF; McBratney AB; Hempel JW; Nauman TW; Brungard CW; Odgers NP POLARIS: A 30-m probabilistic soil series map of the contiguous United States. Geoderma 2016, 274, 54–67.

[48] Daly C; Taylor G; Gibson W The PRISM approach to mapping precipitation and temperature. In Proceedings of the 10th Conference on Applied Climatology, Reno, NV, USA, 20–23 October 1997; American Meteorological Society: Boston, MA, USA, 1997; pp. 10–12.

[49] Stackhouse PW; Zhang T; Westberg D; Barnett AJ; Bristow T; Macpherson B; Hoell JM POWER Release 8 (with GIS Applications) Methodology (Data Parameters, Sources, and Validation—Data Version 8.0.1); NASA Langley Research Center: Hampton, VA, USA, 2018.

[50] Schaaf C; Wang Z MODIS/Terra and Aqua Nadir BRDF-Adjusted Reflectance Daily L3 Global 500 m SIN Grid V006; NASA EOSDIS Land Processes DAAC: Sioux Falls, SD, USA, 2015.

[51] Jiang Y; Xu X; Huang Q; Huo Z; Huang G Assessment of irrigation performance and water productivity in irrigated areas of the middle Heihe River basin using a distributed agro-hydrological model. *Agric. Water Manag*. 2015, 147, 67–81.

[52] McInerney D; Kempeneers P Image (Re-)projections and Merging In Open Source Geospatial Tools; Springer International Publishing: Cham, Switzerland, 2015; pp. 99–127.

[53] Boryan C; Yang Z; Mueller R; Craig M Monitoring US agriculture: The US Department of Agriculture, National Agricultural Statistics Service, Cropland Data Layer Program. Geocarto Int. 2011, 26, 341–358.

[54] Rippey BR The U.S. drought of 2012. *Weather Clim. Extrem*. 2015, 10, 57–64.

[55] Liu X; Jacobs E; Kumar A; Biehl L; Andresen J; Niyogi D The Purdue Agro-climatic (PAC) dataset for the U.S. Corn Belt: Development and initial results. *Clim. Risk Manag*. 2017, 15, 61–72.

[56] Mourtzinis S; Rattalino Edreira JI; Conley SP; Grassini P From grid to field: Assessing quality of gridded weather data for agricultural applications. Eur. J. Agron 2017, 82, 163–172.

[57] White JW; Hoogenboom G; Wilkens PW; Stackhouse PW; Hoel JM Evaluation of Satellite-Based, Modeled-Derived Daily Solar Radiation Data for the Continental United States. *Agron. J*. 2011, 103, 1242.

[58] Xiong W; Holman I; Conway D; Lin E; Li Y A crop model cross calibration for use in regional climate impacts studies. Ecol. Model 2008, 213, 365–380.

[59] Butzen S Corn Seeding Rate Considerations. Available online: https://www.pioneer.com/home/site/us/agronomy/library/corn-seeding-rate-considerations/ (accessed on 27 July 2018).

[60] Liu X; Andresen J; Yang H; Niyogi D Calibration and Validation of the Hybrid-Maize Crop Model for Regional Analysis and Application over the U.S. Corn Belt. *Earth Interact*. 2015, 19.

[61] Combe M; De Wit AJW; Vilà-Guerau De Arellano J; Van Der Molen MK; Magliulo V; Peters W Grain Yield Observations Constrain Cropland CO_2_ Fluxes Over Europe. *J. Geophys. Res*. 2017, 122, 3238–3259.

[62] Challinor AJ; Slingo JM; Wheeler TR; Doblas-Reyes FJ Probabilistic simulations of crop yield over western India using the DEMETER seasonal hindcast ensembles. Tellus A 2005, 57, 498–512.

[63] van Wart J; Kersebaum KC; Peng S; Milner M Estimating crop yield potential at regional to national scales. *Field Crop. Res*. 2013, 143, 34–43.

[64] Wallach D; Goffinet B; Bergez J-E; Debaeke P; Leenhardt D; Aubertot J-N Parameter Estimation for Crop Models. *Agron. J*. 2001, 93, 757.

[65] Doney SC; Yeager S; Danabasoglu G; Large WG; Mcwilliams JC Mechanisms Governing Interannual Variability of Upper-Ocean Temperature in a Global Ocean Hindcast Simulation. *J. Phys. Oceanogr*. 2007, 37.

[66] Cai R; Yu D; Oppenheimer M Estimating the Effects of Weather Variations on Corn Yields using Geographically Weighted Panel Regression. In Proceedings of the Agricultural & Applied Economics Association Annual Meeting, Seattle, WA, USA, 12–14 August 2012.

[67] Greff K; Srivastava RK; Koutnik J; Steunebrink BR; Schmidhuber J LSTM: A Search Space Odyssey. IEEE Trans. Neural Netw. Learn. Syst 2017, 28, 2222–2232. 27411231 10.1109/TNNLS.2016.2582924

[68] Weninger F; Bergmann J; Schuller B Introducing CURRENNT: The Munich Open-Source CUDA RecurREnt Neural Network Toolkit. *J. Mach. Learn. Res*. 2015, 16, 547–551.

[69] Hermans M; Schrauwen B Training and Analyzing Deep Recurrent Neural Networks In Advances in Neural Information Processing Systems 26 (NIPS 2013); NIPS Foundation Inc.: La Jolla, CA, USA, 2013; pp. 1–9.

[70] Kross A; McNairn H; Lapen D; Sunohara M; Champagne C Assessment of RapidEye vegetation indices for estimation of leaf area index and biomass in corn and soybean crops. *Int. J. Appl. Earth Obs. Geoinf*. 2015, 34, 235–248.

[71] Battude M; Al Bitar A; Morin D; Cros J; Huc M; Marais Sicre C; Le Dantec V; Demarez V Estimating maize biomass and yield over large areas using high spatial and temporal resolution Sentinel-2 like remote sensing data. *Remote Sens. Environ*. 2016, 184, 668–681.

[72] Swain S; Wardlow BD; Narumalani S; Rundquist DC; Hayes MJ Relationships between vegetation indices and root zone soil moisture under maize and soybean canopies in the US Corn Belt: A comparative study using a close-range sensing approach. *Int. J. Remote Sens*. 2013, 34, 2814–2828.

[73] Peng C; Deng M; Di L Relationships between Remote-Sensing-Based Agricultural Drought Indicators and Root Zone Soil Moisture: A Comparative Study of Iowa. *IEEE J. Sel. Top. Appl. Earth Obs. Remote Sens*. 2014, 7, 4572–4580.

[74] Gitelson AA; Peng Y; Arkebauer TJ; Schepers J Relationships between gross primary production, green LAI, and canopy chlorophyll content in maize: Implications for remote sensing of primary production. *Remote Sens. Environ*. 2014, 144, 65–72.

[75] Shen Y; Wu L; Di L; Yu G; Tang H; Yu G; Shao Y Hidden Markov Models for Real-Time Estimation of Corn Progress Stages Using MODIS and Meteorological Data. Remote Sens. 2013, 5, 1734–1753.

[76] Sakamoto T; Wardlow BD; Gitelson AA Detecting Spatiotemporal Changes of Corn Developmental Stages in the U.S. Corn Belt Using MODIS WDRVI Data. *IEEE Trans. Geosci. Remote Sens*. 2011, 49, 1926–1936.

[77] Sakamoto T Refined shape model fitting methods for detecting various types of phenological information on major U.S. crops. *ISPRS J. Photogramm. Remote Sens*. 2018, 138, 176–192.

[78] Kang Y; Özdoğan M; Zipper S; Román M; Walker J; Hong S; Marshall M; Magliulo V; Moreno J; Alonso L; How Universal Is the Relationship between Remotely Sensed Vegetation Indices and Crop Leaf Area Index? A Global Assessment. Remote Sens. 2016, 8, 597. [PubMed] 10.3390/rs8070597 PMC6038712 30002923

[79] Koetz B; Baret F; Poilvé H; Hill J Use of coupled canopy structure dynamic and radiative transfer models to estimate biophysical canopy characteristics. *Remote Sens. Environ*. 2005, 95, 115–124.

[80] Bolton DK; Friedl MA Forecasting crop yield using remotely sensed vegetation indices and crop phenology metrics. *Agric. For. Meteorol*. 2013, 173, 74–84.

[81] Kuwata K; Shibasaki R Estimating Corn Yield in The United States with Modis EVI and Machine Learning Methods. *ISPRS Ann. Photogramm. Remote Sens. Spat. Inf. Sci*. 2016, III-8, 131–136.

[82] Du Toit AS; Booysen J; Human JJ Calibration of CERES3 (Maize) to improve silking date prediction values for South Africa. S. Afr. J. Plant Soil 1998, 15, 61–66.

[83] Ali AM; Darvishzadeh R; Skidmore AK Retrieval of Specific Leaf Area from Landsat-8 Surface Reflectance Data Using Statistical and Physical Models. *IEEE J. Sel. Top. Appl. Earth Obs. Remote Sens*. 2017, 10, 3529–3536.

[84] Lymburner L; Beggs PJ; Jacobson CR Estimation of Canopy-Average Surface-Specific Leaf Area Using Landsat TM Data. *Photogramm. Eng. Remote Sens*. 2000, 66, 183–191.

[85] Wolfert S; Ge L; Verdouw C; Bogaardt M-J Big Data in Smart Farming—A review. Agric. Syst 2017, 153, 69–80.

[86] Mourtzinis S; Rattalino Edreira JI; Grassini P; Roth AC; Casteel SN; Ciampitti IA; Kandel HJ; Kyveryga PM; Licht MA; Lindsey LE; Sifting and winnowing: Analysis of farmer field data for soybean in the US North-Central region. *Field Crop. Res*. 2018, 221, 130–141.

[87] Gao F; Anderson M; Daughtry C; Johnson D Assessing the Variability of Corn and Soybean Yields in Central Iowa Using High Spatiotemporal Resolution Multi-Satellite Imagery. Remote Sens. 2018, 10, 1489.

[88] Scharf PC; Kent Shannon D; Palm HL; Sudduth KA; Drummond ST; Kitchen NR; Mueller LJ; Hubbard VC; Oliveira LF Sensor-Based Nitrogen Applications Out-Performed Producer-Chosen Rates for Corn in On-Farm Demonstrations. *Agron. J*. 2011, 103, 1683–1691.

[89] Zhang X; Shi L; Jia X; Seielstad G; Helgason C Zone mapping application for precision-farming: A decision support tool for variable rate application. *Precis. Agric*. 2010, 11, 103–114.

[90] Yan L; Roy DP Conterminous United States crop field size quantification from multi-temporal Landsat data. Remote Sens. Environ 2016, 172, 67–86.

[91] Gao F; Masek J; Schwaller M; Hall F On the blending of the Landsat and MODIS surface reflectance: Predicting daily Landsat surface reflectance. *IEEE Trans. Geosci. Remote Sens*. 2006, 44, 2207–2218.

[92] Claverie M; Ju J; Masek JG; Dungan JL; Vermote EF; Roger J-C; Skakun SV; Justice C The Harmonized Landsat and Sentinel-2 surface reflectance data set. Remote Sens. Environ 2018, 219, 145–161.

[93] Cai Y; Guan K; Peng J; Wang S; Seifert C; Wardlow B; Li Z A high-performance and in-season classification system of field-level crop types using time-series Landsat data and a machine learning approach. *Remote Sens. Environ*. 2018, 210, 35–47.

[94] Siachalou S; Mallinis G; Tsakiri-Strati M A Hidden Markov Models Approach for Crop Classification: Linking Crop Phenology to Time Series of Multi-Sensor Remote Sensing Data. Remote Sens 2015, 7, 3633–3650.

[95] Kussul N; Lavreniuk M; Skakun S; Shelestov A Deep Learning Classification of Land Cover and Crop Types Using Remote Sensing Data. *IEEE Geosci. Remote Sens. Lett*. 2017, 14, 778–782.

[96] Zhong L; Hu L; Yu L; Gong P; Biging GS Automated mapping of soybean and corn using phenology. *ISPRS J. Photogramm. Remote Sens*. 2016, 119, 151–164.

[97] Massey R; Sankey TT; Congalton RG; Yadav K; Thenkabail PS; Ozdogan M; Sánchez Meador AJ MODIS phenology-derived, multi-year distribution of conterminous U.S. crop types. *Remote Sens. Environ*. 2017, 198, 490–503.

[98] Dahal D; Wylie B; Howard D Rapid Crop Cover Mapping for the Conterminous United States. *Sci. Rep*. 2018, 8, 8631. 29872107 10.1038/s41598-018-26284-w PMC5988726

[99] Sakamoto T; Gitelson AA; Arkebauer TJ Near real-time prediction of U.S. corn yields based on time-series MODIS data. *Remote Sens. Environ*. 2014, 147, 219–231.

[100] Veloso A; Mermoz S; Bouvet A; Le Toan T; Planells M; Dejoux J-F; Ceschia E Understanding the temporal behavior of crops using Sentinel-1 and Sentinel-2-like data for agricultural applications. *Remote Sens. Environ*. 2017, 199, 415–426.

[101] Navarro A; Rolim J; Miguel I; Catalão J; Silva J; Painho M; Vekerdy Z Crop Monitoring Based on SPOT-5 Take-5 and Sentinel-1A Data for the Estimation of Crop Water Requirements. Remote Sens. 2016, 8, 525.

[102] Kumar P; Prasad R; Gupta DK; Kumar P; Prasad R; Choudhary A; Mishra VN; Vishwakarma AK; Singh AK; Srivastava PK Comprehensive evaluation of soil moisture retrieval models under different crop cover types using C-band synthetic aperture radar data. Geocarto Int 2018.

[103] Erten E; Lopez-Sanchez JM; Yuzugullu O; Hajnsek I Retrieval of agricultural crop height from space: A comparison of SAR techniques. *Remote Sens. Environ*. 2016, 187, 130–144.

