# Supplementary material for: Utilizing Collocated Crop Growth Model Simulations to Train Agronomic Satellite Retrieval Algorithms
Source: Remote Sens (Basel). Author manuscript; Available in PMC 2019 Jan 28. (PMC6349260; doi:10.3390/rs10121968)
Supplement: S001 [file NIHMS1003441-supplement-S001.pdf]

Supplementary Materials for "Utilizing Collocated Crop Growth Model Simulations to Train Agronomic Satellite Retrieval Algorithms"

Table S1: Mapping of Phenology BLTSM-predicted maize stages to APSIM and USDA NASS maize stages [1]

BLSTM Stages	APSIM Stages
Pre- and Post- Major Growth	0 (No Growth), 1 (Germinating), 2 (Emerging), 9 (Maturing), 10 (MaturityToHarvestRipe), 11 (ReadyForHarvesting)
	**Note**: Stages 9, 10, and 11 generally last for only a day each in the APSIM simulations we performed and it is acceptable to group them in this stage due to the inherent variability in when the farmer actually performs the harvest
Emergence to Floral Initiation	3 (Juvenile), 4 (Photosensitive Period)
Floral Initiation to Silking	5 (Leaf Appearance), 6 (FlagLeafToFlowering)
Silking to Start Grain Fill	7 (FloweringToGrainFilling)
Start Grain Fill to End Grain Fill	8 (GrainFilling)

Empirical Orthogonal Function-Based (EOF) Model Validation Analysis

1. Methods

In order to validate the spatial performance of the model and separate it from its interannual temporal performance, an empirical orthogonal function-based (EOF) model validation analysis is conducted [2]. The EOF analysis decomposes the data into temporal and spatial components as

$$Y[x, y, t] = \mu[t] + \sum_i PC_i[t] * EOF_i[x, y], \quad (1)$$

where $\mu[t]$ is the mean interannual yield time series calculated over the US Corn Belt, $PC_i[t]$ are the i temporal principal components calculated over the US Corn Belt, and $EOF_i[x, y]$ are the corresponding spatial EOF patterns. In order to focus on the validation of the spatial variability, the procedure from [2] is modified by calculating the principal components only on the ground-truth NASS yield data and projecting both the actual and simulated yields onto these same principal components. This ensures that the projection to obtain the spatial EOF patterns is the same for both the actual and simulated yields, allowing the comparison between the simulated and actual patterns to focus solely on the spatial performance of the model. As EOF analysis requires data for all years from a county to be available, counties where the NASS ground truth yields were not available for all study years were removed prior to performing the EOF analysis. It has been observed previously [3] that many counties have at least one year of yield data missing, causing these counties to be removed when performing analysis that requires all years to be present. Once the actual and simulated spatial EOF patterns are obtained, the ability of the model to reproduce the actual spatial patterns of the most significant EOFs is assessed through scatterplots and associated R^2 and RMSE values.

2. Results

The results of the EOF-based analysis to assess the spatial performance of the model are shown in Figure S4 for the clusterless calibration. Figure S4 shows the 4 most significant spatial EOF patterns of the ground truth USDA county yields, as well as corresponding scatterplots of the predicted versus actual EOF components. The displayed spatial patterns provide information about the modes of spatial yield variability across the US Corn Belt. The results in the corresponding scatterplots show the extent to which each mode can be reproduced by the model. Specifically, the scatterplots show that the first three EOF

components, which represent 86% of the total variability in the actual yields, can be reproduced with R^2 values above 0.4.

Supplementary Figures

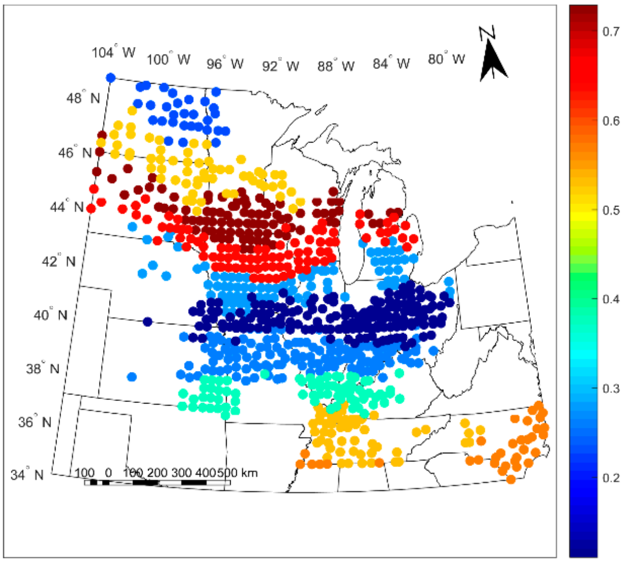

Figure S1a: LOO yield prediction R^2 values by cluster for 10 cluster weather-based clustering calibration

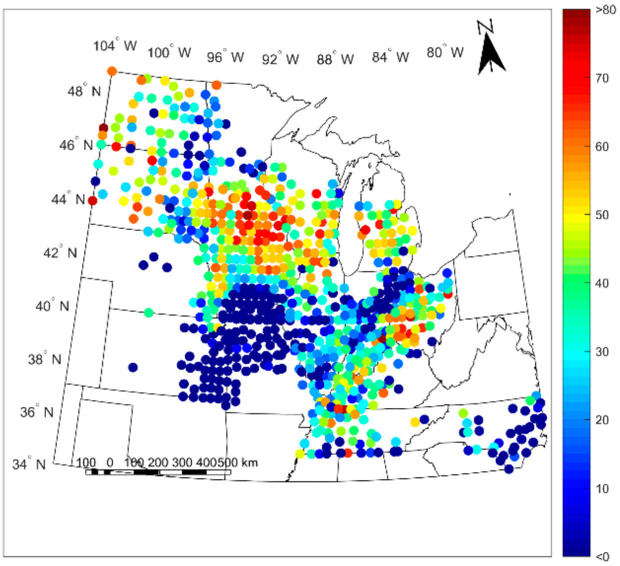

Figure S1b: LOO yield prediction ESTD [%] values averaged for each county for the 10 cluster weather-based clustering calibration

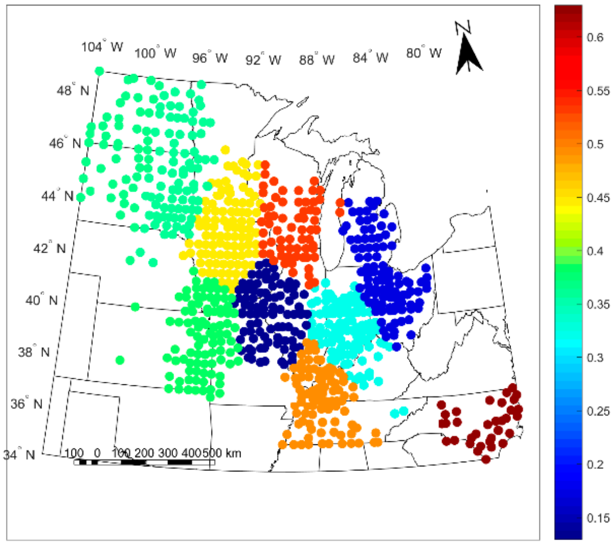

Figure S2a: LOO yield prediction R^2 values by cluster for 10 cluster geographic-based clustering calibration

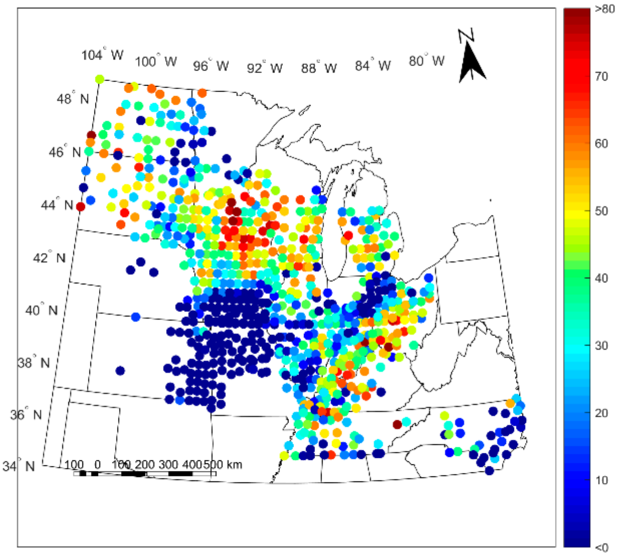

Figure S2b: LOO yield prediction ESTD [%] values averaged for each county for the 10 cluster geographic-based clustering calibration

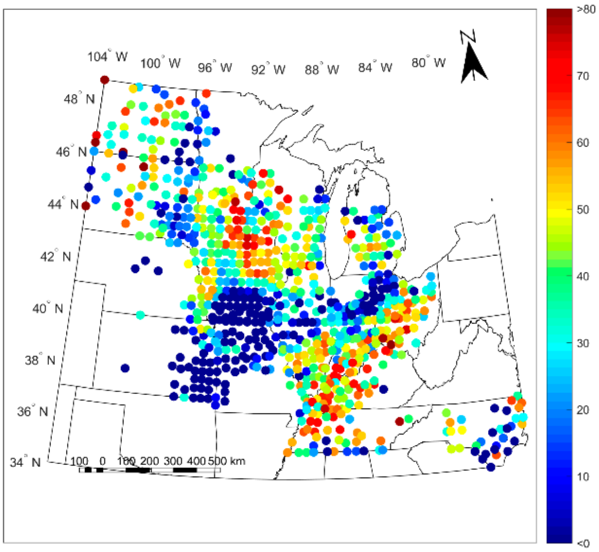

Figure S3: LOO yield prediction ESTD [%] values averaged for each county for the clusterless calibration

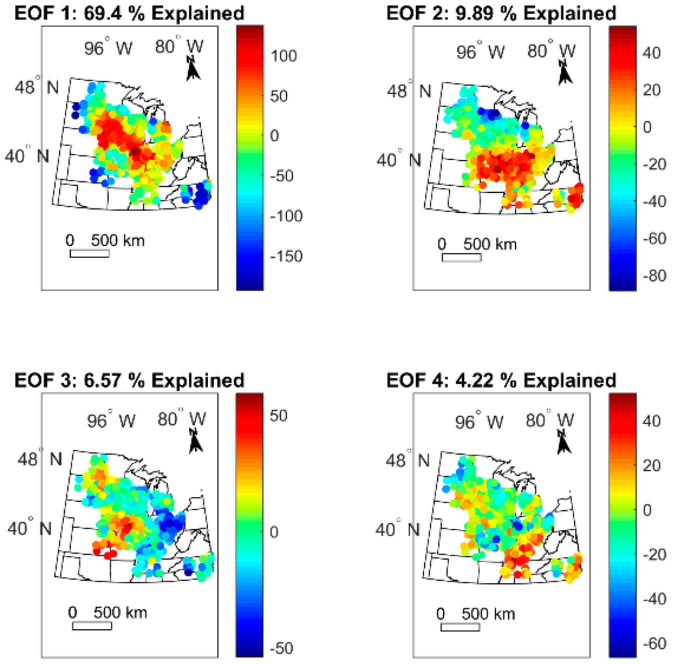

(a)

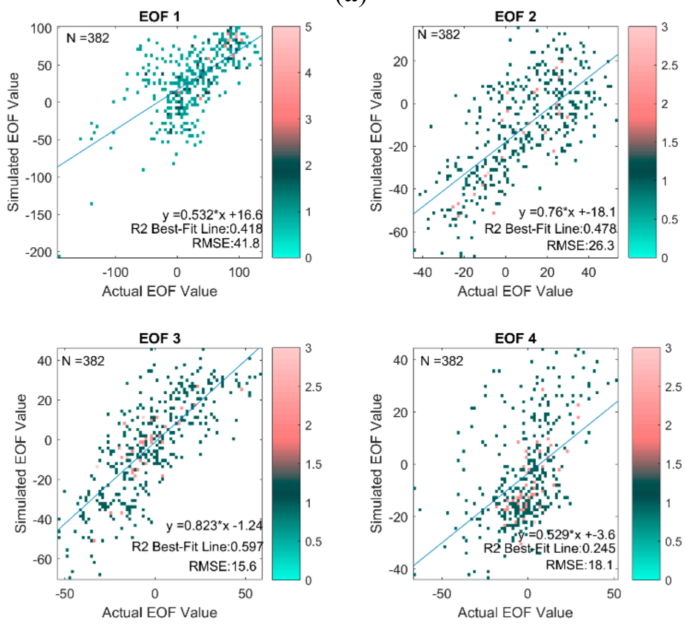

(b)

Figure S4: EOF-based spatial performance analysis for clusterless calibration across the entire US Corn Belt. **(a)** Spatial EOF pattern of the 4 most significant ground truth USDA county spatial EOF patterns. The percentage of the spatial variability of the actual crop yield explained by each EOF component is shown in the heading for each column. **(b)** Scatterplots between the crop model predicted EOF spatial values and the actual EOF spatial values. Colorbars on the scatterplots indicate number of points at a particular pixel in the scatterplot.

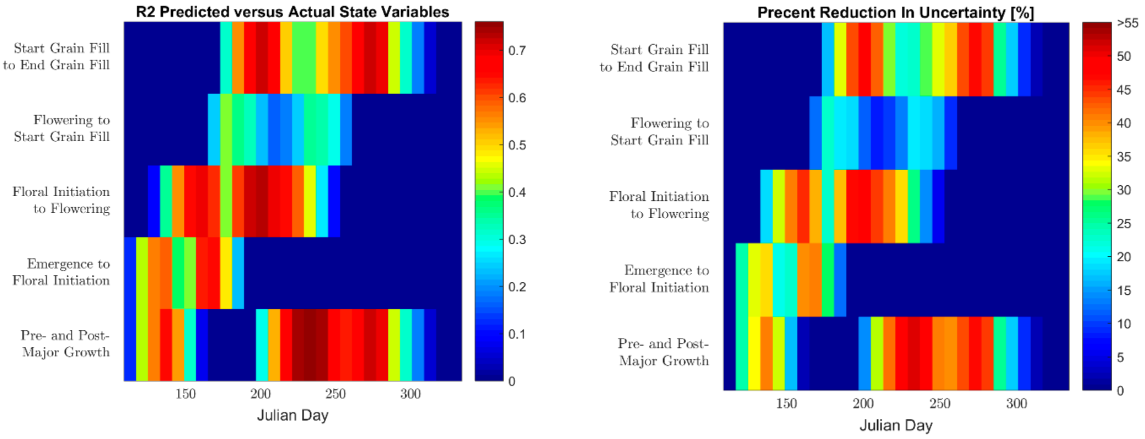

(a) (b)

Figure S5: (a) CV R^2 and (b) CV PRU phenological state variable prediction results for clusterless calibration over entire US Corn Belt

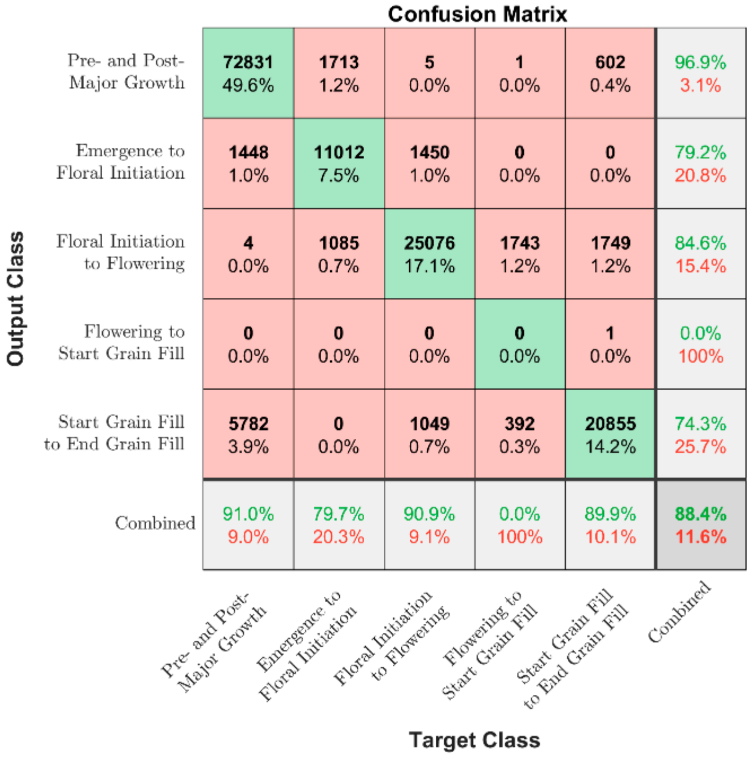

Figure S6: CV phenological state variable confusion matrix for clusterless calibration over entire US Corn Belt

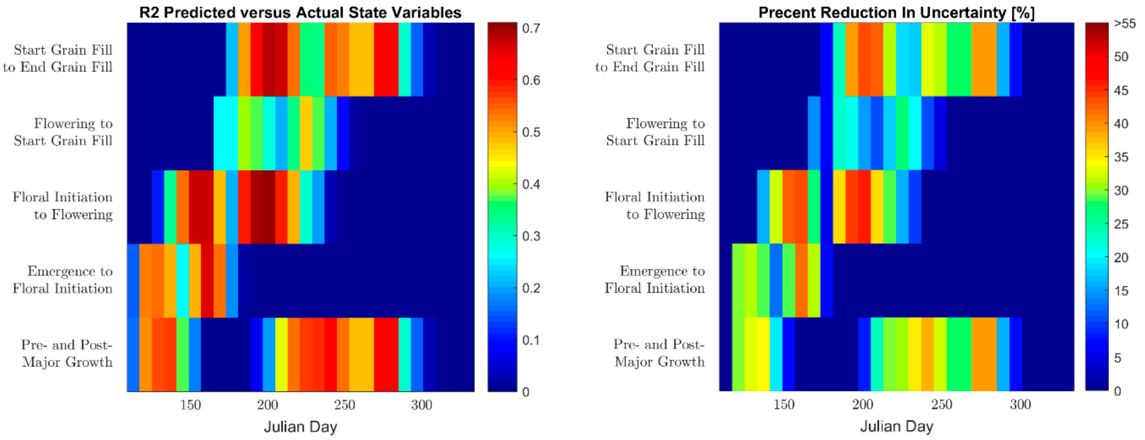

Figure S7: (a) CV R^2 and (b) CV PRU phenological state variable prediction results in selected weather-based clusters

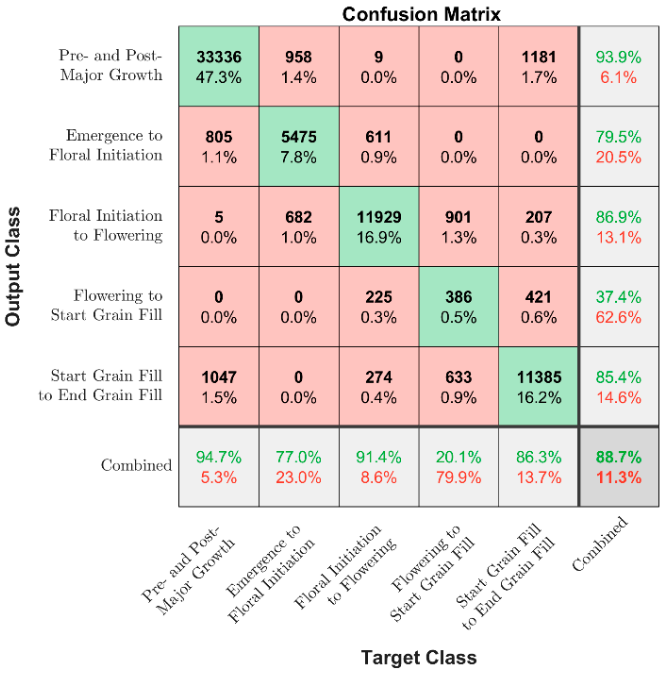

Figure S8: CV phenological state variable confusion matrix in selected weather-based clusters

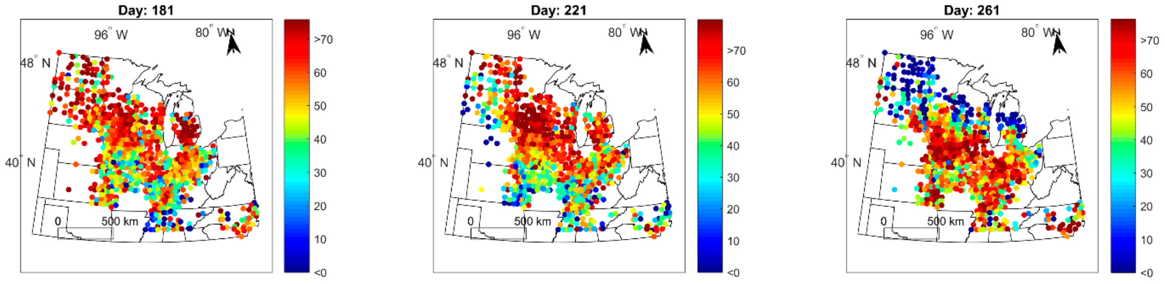

Figure S9a: ESTD Values for retrieved Leaf Area Index for clusterless calibration over entire US Corn belt

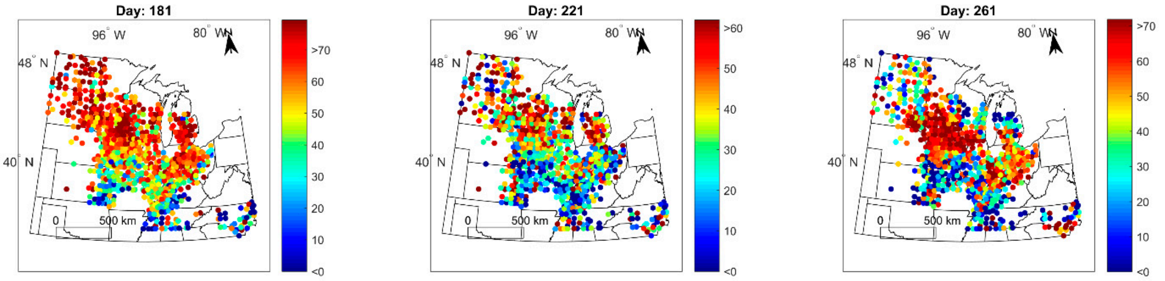

Figure S9b: ESTD Values for retrieved Aboveground Biomass for clusterless calibration over entire US Corn belt

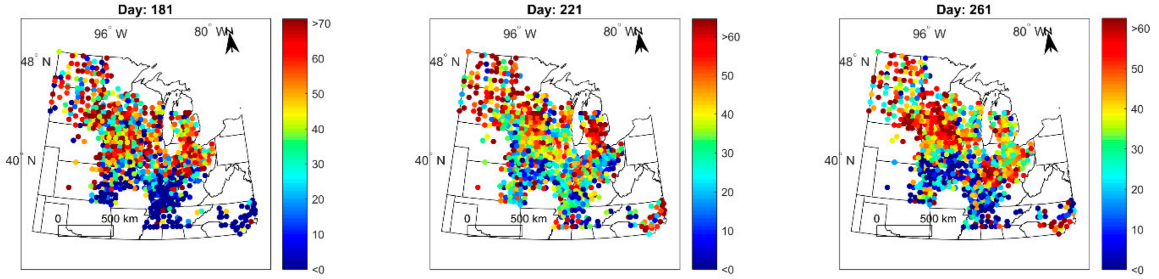

Figure S9c: ESTD Values for retrieved Harvested Organ Biomass for clusterless calibration over entire US Corn belt

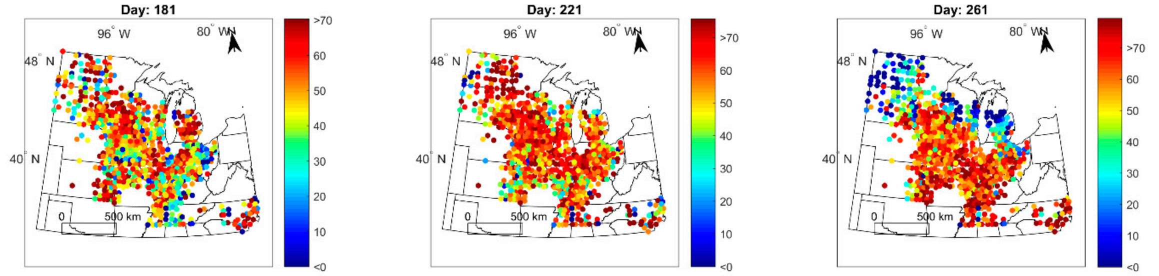

Figure S9d: ESTD Values for retrieved Leaf Nitrogen Biomass for clusterless calibration over entire US Corn belt

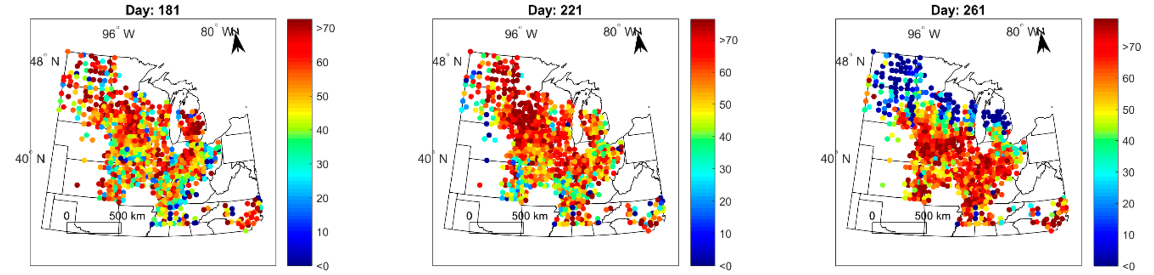

Figure S9e: ESTD Values for retrieved Total Leaf Biomass for clusterless calibration over entire US Corn belt

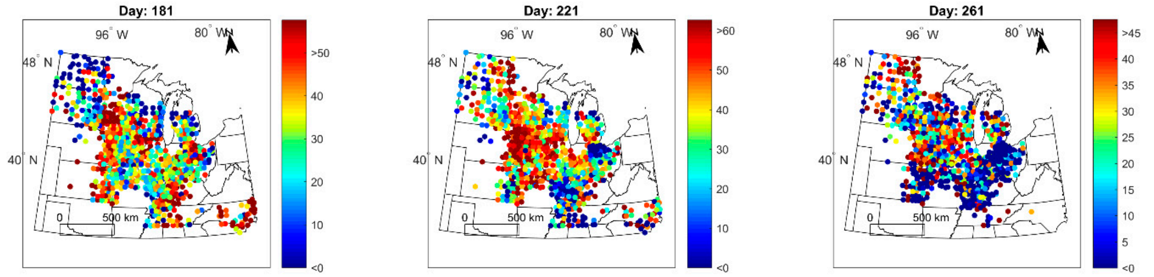

Figure S9f: ESTD Values for retrieved Specific Leaf Area for clusterless calibration over entire US Corn belt

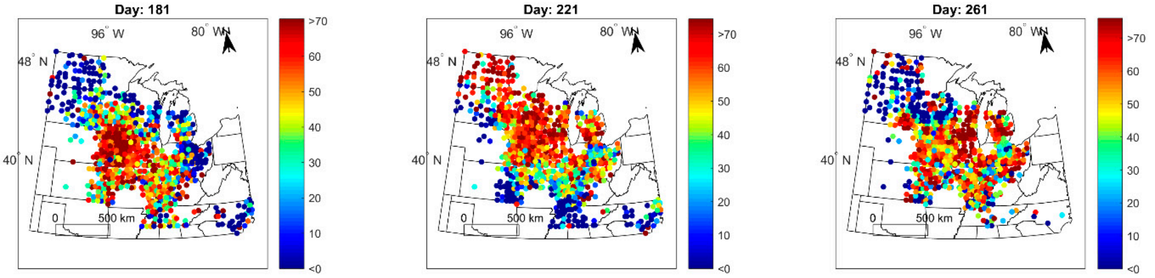

Figure S9g: ESTD Values for retrieved Leaf Nitrogen Percentage for clusterless calibration over entire US Corn belt

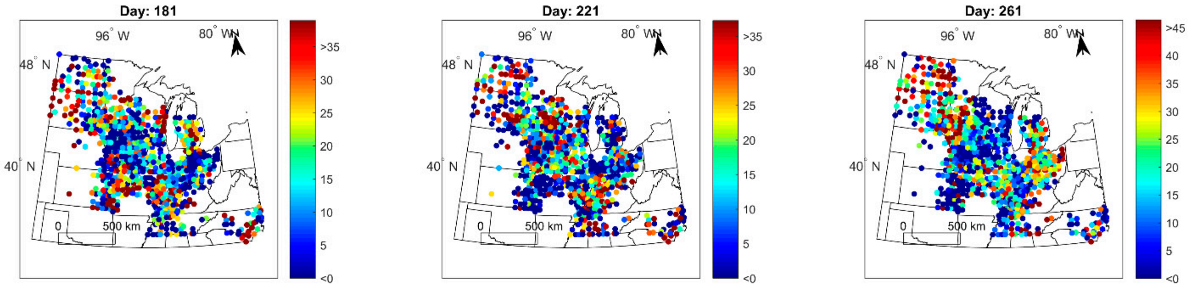

Figure S9h: ESTD Values for retrieved Soil Moisture 0 – 5 cm for clusterless calibration over entire US Corn belt

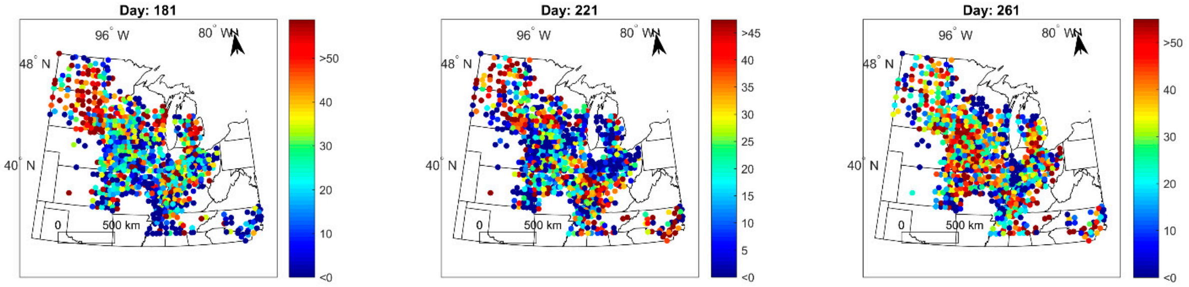

Figure S9i: ESTD Values for retrieved Soil Moisture 5 – 15 cm for clusterless calibration over entire US Corn belt

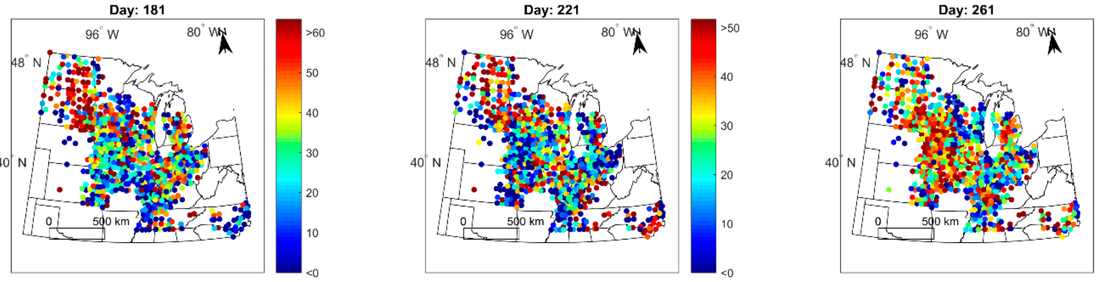

Figure S9j: ESTD Values for retrieved Soil Moisture 15 – 30 cm for clusterless calibration over entire US Corn belt

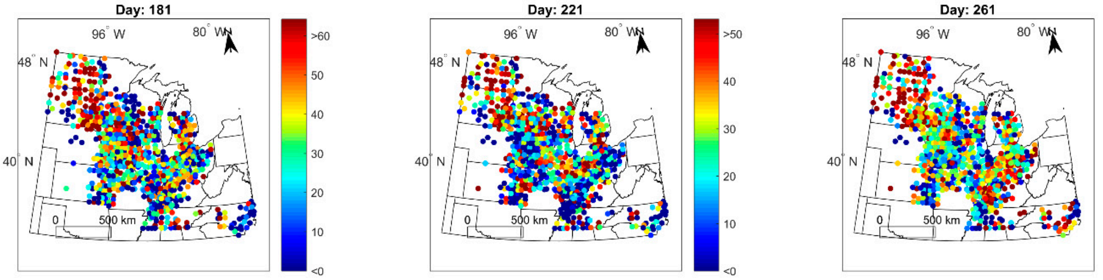

Figure S9k: ESTD Values for retrieved Soil Moisture 30 – 60 cm for clusterless calibration over entire US Corn belt

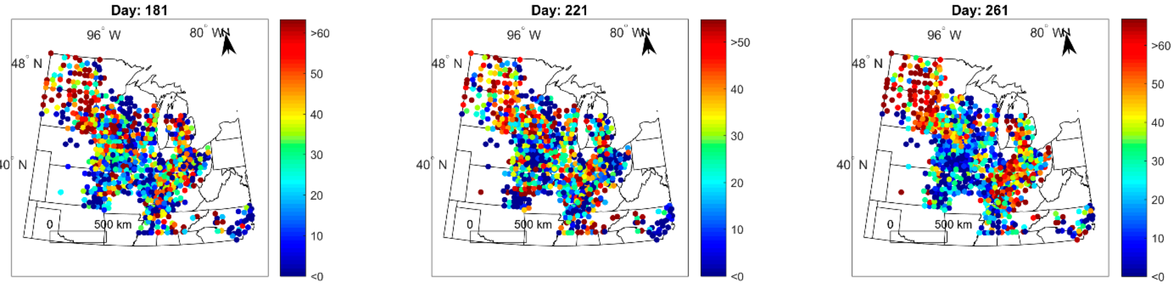

Figure S9l: ESTD Values for retrieved Soil Moisture 60 – 100 cm for clusterless calibration over entire US Corn belt

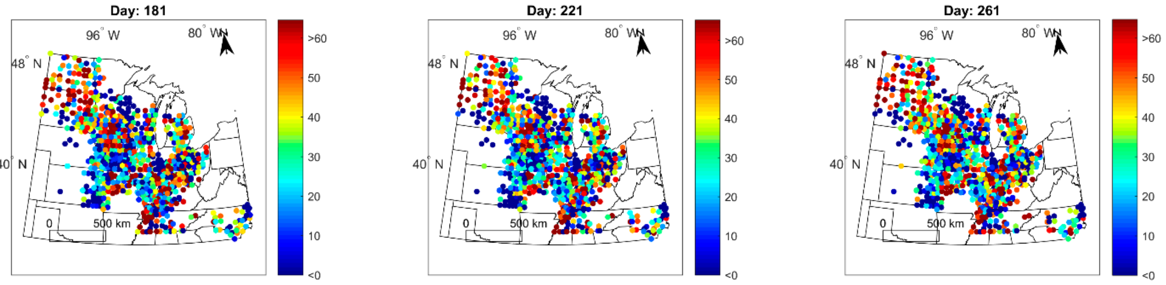

Figure S9m: ESTD Values for retrieved Soil Moisture 100 – 200 cm for clusterless calibration over entire US Corn belt

References

1. Brown, H. E.; Teixeira, E. I.; Huth, N. I.; Holzworth, D. P. *The APSIM Maize Model*;
2. Doney, S. C.; Yeager, S.; Danabasoglu, G.; Large, W. G.; Mcwilliams, J. C. Mechanisms Governing Interannual Variability of Upper-Ocean Temperature in a Global Ocean Hindcast Simulation.

2007, doi:10.1175/JPO3089.1.

3. Cai, R.; Yu, D.; Oppenheimer, M. Estimating the Effects of Weather Variations on Corn Yields using Geographically Weighted Panel Regression. In *Agricultural & Applied Economics Association Annual Meeting*; Seattle, Washington, 2012.
